# Radular force performance of stylommatophoran gastropods (Mollusca) with distinct body masses

**DOI:** 10.1038/s41598-021-89892-z

**Published:** 2021-05-18

**Authors:** Wencke Krings, Charlotte Neumann, Marco T. Neiber, Alexander Kovalev, Stanislav N. Gorb

**Affiliations:** 1 Department of Mammalogy and Palaeoanthropology, Center of Natural History (CeNak), Universität Hamburg, Martin-Luther-King-Platz 3, 20146 Hamburg, Germany; 2 Department of Animal Diversity, Center of Natural History (CeNak), Universität Hamburg, Martin-Luther-King-Platz 3, 20146 Hamburg, Germany; 3 Department of Functional Morphology and Biomechanics, Zoological Institute, Christian-Albrechts-Universität zu Kiel, Am Botanischen Garten 9, 24118 Kiel, Germany

**Keywords:** Zoology, Experimental organisms

## Abstract

The forces exerted by the animal’s food processing structures can be important parameters when studying trophic specializations to specific food spectra. Even though molluscs represent the second largest animal phylum, exhibiting an incredible biodiversity accompanied by the establishment of distinct ecological niches including the foraging on a variety of ingesta types, only few studies focused on the biomechanical performance of their feeding organs. To lay a keystone for future research in this direction, we investigated the in vivo forces exerted by the molluscan food gathering and processing structure, the radula, for five stylommatophoran species (Gastropoda). The chosen species and individuals have a similar radular morphology and motion, but as they represent different body mass classes, we were enabled to relate the forces to body mass. Radular forces were measured along two axes using force transducers which allowed us to correlate forces with the distinct phases of radular motion. A radular force quotient, AFQ = mean Absolute Force/bodymass^0.67^, of 4.3 could be determined which can be used further for the prediction of forces generated in Gastropoda. Additionally, some specimens were dissected and the radular musculature mass as well as the radular mass and dimensions were documented. Our results depict the positive correlation between body mass, radular musculature mass, and exerted force. Additionally, it was clearly observed that the radular motion phases, exerting the highest forces during feeding, changed with regard to the ingesta size: all smaller gastropods rather approached the food by a horizontal, sawing-like radular motion leading to the consumption of rather small food particles, whereas larger gastropods rather pulled the ingesta in vertical direction by radula and jaw resulting in the tearing of larger pieces.

## Introduction

The typical force exerted by feeding organs is a useful parameter indicating specializations to distinct food types, as it correlates with the food spectrum (see e.g.^1^; for a review for stress-related puncture mechanics, see^2^). This topic had been studied quite intensively in vertebrates (for a summary of the relevant literature, see^3^): bite force analyses had been performed on mammals (e.g.^4–8^), reptiles (e.g.^9–12^), fish (e.g.^13–15^), and birds (e.g.^16,17^). Even though the majority of animal species belong to the invertebrates, unfortunately fewer work focused on the forces exerted by their structures involved in either gathering or acquiring food due to the difficulties of an experimental set-up for studies of small structures. Exceptions and pioneers in this field are studies performed on representatives of Arthropoda: spiders, crustaceans, scorpions, and insects^18–24^.

For molluscs, even though they represent the second specious animal group^25^ with around 80,000 recent species only within the Gastropoda^26^, only a few studies approached the forces exerted by their feeding organ, the radula. Since the species belonging to this animal phylum occupy almost any marine, freshwater or terrestrial environment and established extremely varied ecological niches^27^, accompanied with feeding on a wide range of food sources with various mechanical properties, mollusc trophic specializations are of very high interest for evolutionary biologists. Their radula, one important molluscan autapomorphy and the interface between the organism and its ingesta (food, minerals), is highly diverse and offers an immense opportunity to study the structural adaptations enabling feeding on distinct ingesta types^28^.

The radula consists of a thin, chitinous membrane with rows of embedded, sometimes mineralized teeth which is supported by thick, underlying odontophoral cartilages and moved by numerous muscles of the buccal mass. The sometimes highly complex radular motion (see e.g.^29–34^) brings the tooth cusps in contact with the ingesta leading to the tearing, cutting, and gathering of food.

As the teeth are involved as actual organism-ingesta interface, usually the shape of teeth or overall morphology of the radula had been examined and related to the ingesta (e.g.^35–49^; also examined for the tooth anchorage:^50^). In the context of phenotypic plasticity, different shapes of radular teeth as an answer to shifts of the ingesta have also been studied^40,51–61^. Sometimes these analyses are complemented by material property estimations of teeth^33,62–73^. Additionally, ingesta consumption, grazing activity, food choice experiments, and fecal analyses for diverse gastropod species have been investigated relating the gastropods with their preferred food, the abundance of food or other parameters of the microhabitat (^74–80^; for comprehensive reviews on diet of Heterobranchia, see^81,82^).

The majority of previous studies have focused on the radular teeth themselves, but the forces exerted by this organ or its biomechanical performance have unfortunately only been investigated in a few papers devoted (1) to the feeding force calculations (^83^; or force calculations for radula-inspired gripping devices:^84^), (2) to the experiments revealing the forces needed to remove algae^85,86^, or (3) to the first in vivo experiment performed on a single mollusc species^87^.

Here, before the broad topic of trophic specialization in molluscs can be approached and, to lay a keystone for further studies, we investigated the in vivo radular forces while foraging for five different stylommatophoran species using force transducers following the protocol of^87^. As the radular motion or radular morphology could potentially influence the forces exerted by the feeding organ we first selected species with a similar radular type (isodont) and similar radular motion to get a good impression on radular forces without being confronted with overflowing radular diversity, which could make results less comparable or prone to artefacts. Additionally, since the chosen gastropods however represent distinct body mass classes, we tested if and to which extend the chosen stylommatophoran specimens follow common laws and predictions for scaling of force and body mass.

## Material and methods

For force measurements we have chosen five stylommatophoran species (Gastropoda: Heterobranchia) that were easy to obtain, as they are either often kept as pets or could be collected easily around Hamburg. Additionally, all these species possess a similar radular type with numerous, similar shaped (isodont), and small teeth, thus the direct influence of the tooth morphology on the forces produced is probably rather small. Also, stylommatophoran gastropods show a rather similar radular foraging motion, which can be described as a licking motion (for details on motion and radular type see e.g.^34,87–93^). There are surely differences in motion between species, because the arrangement or thickness of radular muscles might differ. Additionally, individual gastropods of the same species might also prefer a slightly different feeding motion, but the broad cycles of the pro- and retraction are comparable.

**Figure 1:**
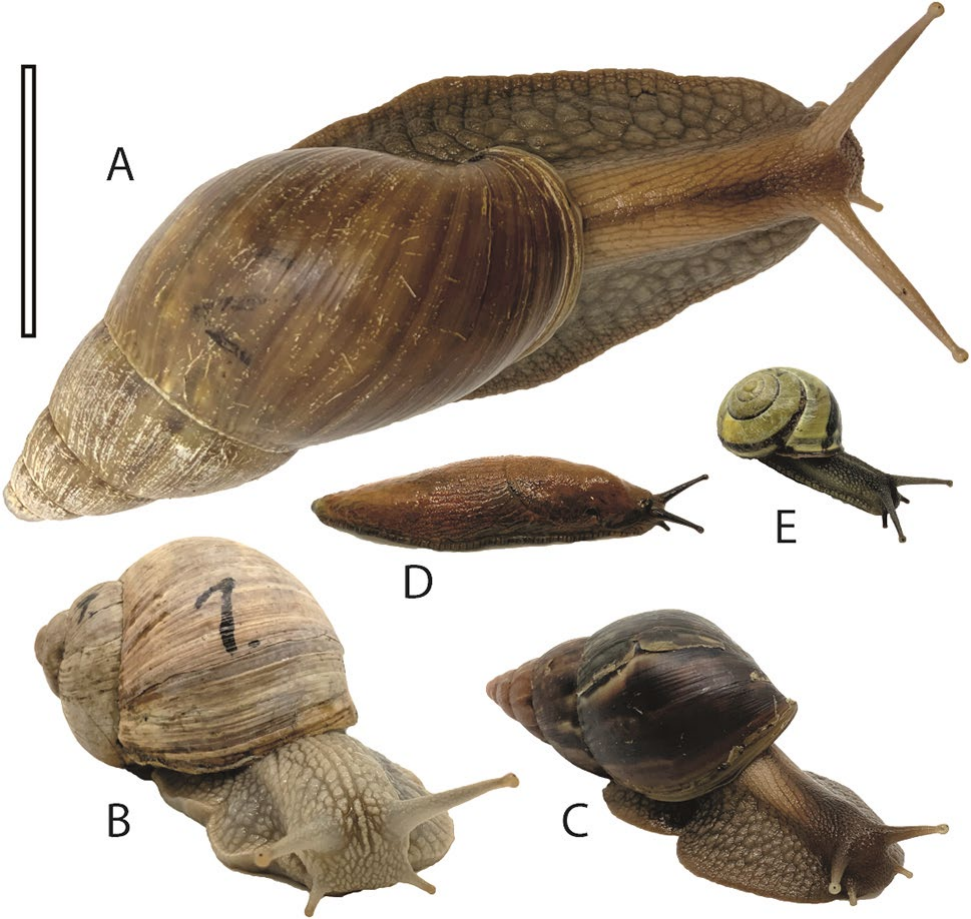
Mollusc species used in this study. (**A**) Mature *Lissachatina fulica*. (**B**) *Helix pomatia*. (**C**) Immature *Lissachatina fulica.* (**D**) *Arion vulgaris*. (**E**) *Cepaea nemoralis*. Scale bar = 4 cm.

We have chosen the following gastropods, since they can be sorted to three distinct body mass classes (see Fig. 1 and below): mature *Lissachatina fulica* (Bowdich, 1822), mature *Cepaea nemoralis* (Linnaeus, 1758)*,* mature *Cepaea hortensis* (Müller, 1774)*,* mature *Helix pomatia* Linnaeus, 1758, and mature *Arion vulgaris* Moquin-Tandon, 1855. Since no adult stylommatophoran species with a body mass between mature *Helix* and mature *Lissachatina fulica* is easily obtainable, we selected immature *Lissachatina fulica* (one juvenile stage) for experiments. Overall, 24 individuals were used; individuals of *L. fulica* were received from private animal breeders, the other species were collected in Hamburg, Germany, in May 2020. Species identification is based on the relevant literature.

The selected gastropods species were assigned to three different body mass classes: (A) large sized animals (whole body mass: 75–105 g) = mature *L. fulica* [N = 5 individuals]; (B) medium sized animals (whole body mass: 10–38 g) = *H. pomatia* and immature *L. fulica* [N = 10 individuals]; (C) small sized animals (whole body mass: 0.7–3.6 g) = *C. nemoralis*, *C. hortensis*, *A. vulgaris* [N = 9 individuals]); and cohorts (mature *L. fulica* [N = 5 individuals], immature *L. fulica* [N = 9 individuals], mature *H. pomatia* [N = 1 individual], mature *C. nemoralis* [N = 2 individuals], mature *C. hortensis* [N = 1 individual], mature *A. vulgaris* [N = 6 individuals]). Before each experiment, individuals were weighed (body mass with shell) (see Supplementary Table 1).

**Figure 2:**
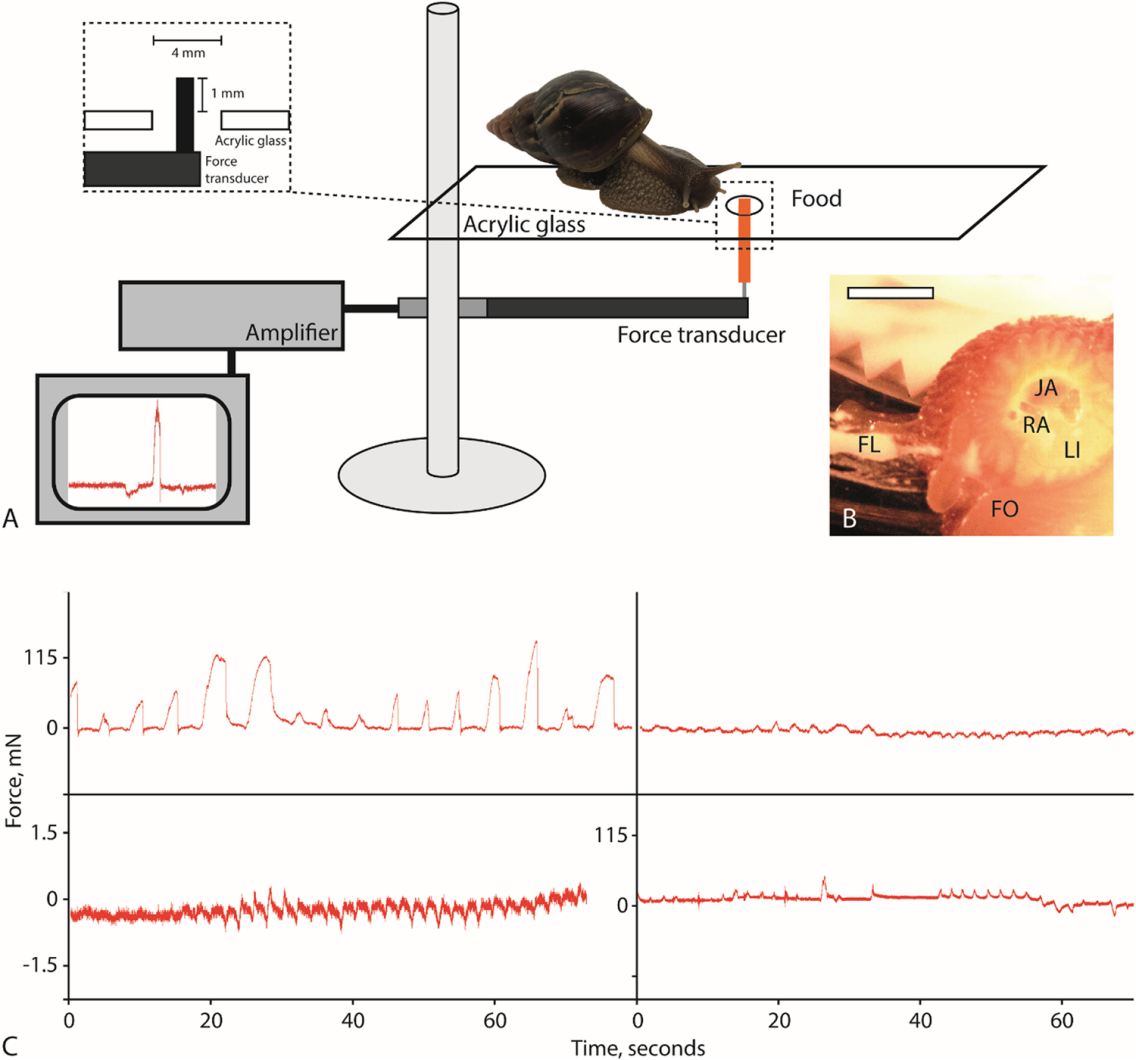
Experimental set-up (drawn with Adobe Illustrator CS 6 and modified from^87^) and characteristic radular force measurements. (**A**) Gastropods were placed on an acrylic platform with a hole of 2 mm diameter. The sliced food (e.g. carrot), glued to a needle, was firmly mounted to a force transducer connected with an amplifier and computer-based data acquisition and processing system. The food was stuck through the hole, so that animals could feed on it without involving their foot. (**B**) Image of the mouth opening taken through the glass platform, scale bar = 1 cm. (**C**) Characteristic radular force measurement curves, mN, of mature *Lissachatina fulica*, (above) and *Arion vulgaris* (below); left side: vertical direction (positive peaks = pulling up, negative peaks = pushing down), right side: horizontal direction (positive peaks = posterior direction, negative peaks = anterior direction). JA = jaw, FL = flour, FO = foot, LI = lip, RA = radula.

Forces exerted by the radula were measured following the protocol of^87^. Snails were placed on an acrylic platform with a small hole of 4 mm diameter. The platform was attached to a laboratory jack so that the height could be adjusted. Food stripes (sliced to pieces of 3 [width] × 2 [length] × 20 or 40 [height] mm; either carrot (for all species except *A. vulgaris*) or fresh strawberry (for *A. vulgaris*), depending on the specific preference of the species, were glued to a needle, which was mounted onto a force transducer FORT-10 (World Precision Instruments, Sarasota, FL, USA) and stuck through the hole so that the snail could feed on it, but without the involvement of the foot (Fig. 2). A 1000 g sensor was used for mature *L. fulica*, for all other individuals a 25 g sensor was used. Forces could only be measured in either vertical or horizontal direction, but not in both simultaneously. Thus the experimental set-up was remodeled to receive data for both directions (see also^87^). The force transducers were connected to an amplifier (Biopac System, Inc., CA, USA) and a computer-based data acquisition and processing system (AcqKnowledge™, Biopac Systems, Inc., v.3.7.0.0, World Precision Instruments, Sarasota, FL, USA). Not all force peaks were analyzed in AcqKnowledge™ due to the large sample size, but about 30 maximal and minimal force peaks were evaluated per feeding unit, which is the time needed for eating up the part of the carrot or strawberry that was not coated with glue. For the detailed quantity of evaluated force peaks per species, individual, and direction see Supplementary Table 1; overall, 4407 force peaks were analyzed (see Fig. 2C for characteristic force peaks). Forces were either sorted to direction (negative force values = pushing, positive force values = pulling) or the absolute values summarized as Absolute Force (thus, regardless of the direction). Force values were corrected for mean body mass with shell to receive Relative Force I (force/mean body mass with shell, mN/g). Mean and standard deviations were calculated and all statistical analyses were performed with the program JMP Pro 14 (SAS Institute Inc., Cary, NC, 1989–2007) comparing the exerted forces between (a) the distinct body mass classes, (b) the cohorts, (c) the directions, (d) the individual animals. A Shapiro–Wilk test to test distribution was carried out and since data is non parametric a Kruskal–Wallis test was executed. For linear regression of Absolute Force and Relative Force I *versus* whole body mass, the mean and the values were displayed on logarithmic axes with excel 2013 (Microsoft Corporation, Redmond, USA) and trend lines were generated. A Radular Force Quotient, AFQ = mean Absolute Force/Bodymass^0.67^ and a Relative Force Quotient, RFQ = mean Relative Force I/Bodymass^−0.33^, were determined. Additionally, such quotients were also determined for Forces sorted to direction/Bodymass^−0.33^ and for mean Relative Force I sorted to direction/Bodymass^−0.33^.

Detailed radular motion, while feeding on a flat surface was documented with a Keyence VHX-500 digital microscope (KEYENCE, Neu-Isenburg, Germany) by placing the individual on an acrylic platform, providing flour paste as food (see also^34,87^). The behavior, while foraging on a carrot, was documented with an iPad Pro (11 zoll; Apple Inc., Cupertino, USA) equipped with a 12 megapixel wide angle lens with 30 frames per second (see Supplementary Videos 1 and 2). Videos were cut and cropped with Adobe Premiere Pro 2020 (Adobe Inc., San Jose, USA).

Some animals (see Supplementary Fig. 8) were either killed by brief boiling (shelled animals) or by placing them in carbonated water (slugs). They were preserved in 70% EtOH and inventoried in the malacological collection of the Zoological Museum Hamburg (ZMH) of the Centrum für Naturkunde (CeNak); *Cepaea nemoralis*: ZMH 154748/8, *Helix pomatia*: ZMH 154749/1, *Lissachatina fulica*: ZMH 154751/2, *Cepaea hortensis*: ZMH 154754/1, *Arion vulgaris*: ZMH 154747/12.

The shells of dead specimens were removed and the soft parts were weighed to receive body mass without shell. Forces were corrected for body mass without shell to receive Relative Force II (force/body mass without shell, mN/g). To estimate the mass of the entire buccal mass (BRJ), the radula and jaw (RJ), and the buccal mass musculature (B) these specimens were dissected which was documented with a Keyence VHX-500 digital microscope (KEYENCE, Neu-Isenburg, Germany). The BRJ was first extracted, freed from surrounding tissue (see Supplementary Fig. 7) and weighed in wet condition with an accuracy weighing machine (Sartorius Cubis, MSE, Sartorius AG, Göttingen, Germany). Subsequently the radula and jaw (RJ) were separated from the buccal mass musculature (B) manually; RJ and B were weighed in wet condition. B and RJ were then dried for one week and weighed again to obtain data on dry mass. Forces were corrected for dry B to receive Relative Force III (force/dry B mass, mN/mg) and for dry RJ to receive Relative Force IV (force/dry RJ mass, mN/mg).

For scanning electron microscope (SEM) images radulae and jaws were rewetted and cleaned with proteinase K digesting food particles according to the protocol of^94^, followed by a short ultrasonic bath. Structures were mounted on SEM stubs, coated with palladium and visualized with a Zeiss LEO 1525 (One Zeiss Drive, Thornwood, USA). Radular length, width, and area could be calculated. Forces were corrected for radular area to receive Relative Force V (force/radular area, mN/mm^2^).

## Results

### Radula and its teeth

All analyzed species possess an isodont radula (Supplementary Figs. 3–6). *Lissachatina fulica* displays ~ 84 teeth per row (no central tooth, 16 lateral and 26 marginal teeth on each side), *Helix pomatia* ~ 87 teeth per row (one central tooth, 23 lateral and 20 marginal teeth on each side), *Arion vulgaris* ~ 75 teeth per row (one central tooth, 16 lateral and 21 marginal teeth on each side), *Cepaea nemoralis* and *C. hortensis* ~ 51 teeth per row (one central tooth, 12 lateral and 13 marginal teeth on each side). The jaws of all species are thick, curved, chitinous plates with ribs (i.e. odontognathous).

### Radular motion

Video footage reveals the radular motion and feeding behaviour (see Supplementary Videos 1 and 2). While feeding, the radula is pushed simultaneously in ventral (vertical down) and anterior (horizontal anterior) direction, before the organ is finally pulled in dorsal (vertical up) and posterior (horizontal posterior) direction and the mouth is closed (see also^87^). With the first part of the motion the radula loosens food items from the ground and collects particles, which are transported into the mouth opening in the latter phase of the feeding action. When feeding on larger ingesta (e.g. a piece of carrot; see Supplementary Video 1), the anterior part of the radula and the jaw act in concert as counter bearing squeezing and pulling the ingesta. When comparing the feeding behaviours of different individuals we can see that the large sized individuals can completely enclose the carrot piece with their lips, resulting in a dragging on the carrot in vertical direction, tearing large pieces, whereas the small and medium sized individuals are not as comfortable with this due to the small dimension of their mouth. These individuals usually nibble on edges of the item, cutting and slicing smaller pieces in rather horizontal direction employing their radula like a saw, sometimes involving additionally the foot as a clamp (Supplementary Video 2). They also drag the food in ventral direction, but this behaviour is not as pronounced and is not as forcefully as in the smaller specimens (Fig. 4). All gastropods were able to consume the food items offered, but small sized individuals needed to invest approximately 800–900% and medium sized ones 400–500% more time to consume the similar sized food items than large sized gastropods.

### Force measurements

**Figure 3:**
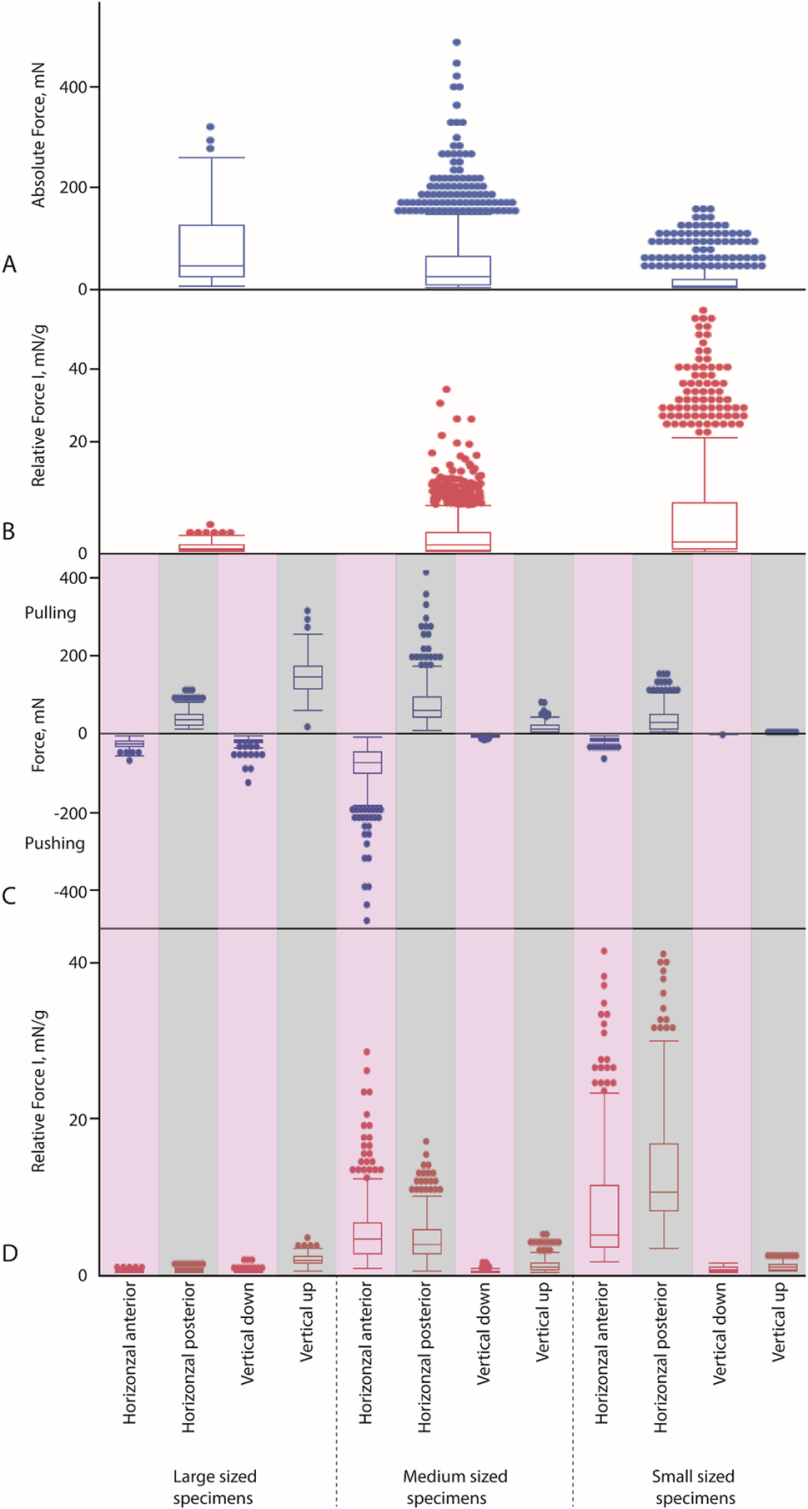
Absolute Force (blue boxplots) and Relative Force I (red boxplots) for distinct body mass classes (large-, medium-, and small-sized gastropods). (**A**) Absolute Force (regardless of the direction of measurement). (**B**) Relative Force I (regardless of the direction of measurement). (**C**) Absolute Force sorted to directions) (**D**) Relative Force I sorted to directions. Pink = pushing of radula, grey = pulling of radula.

**Figure 4:**
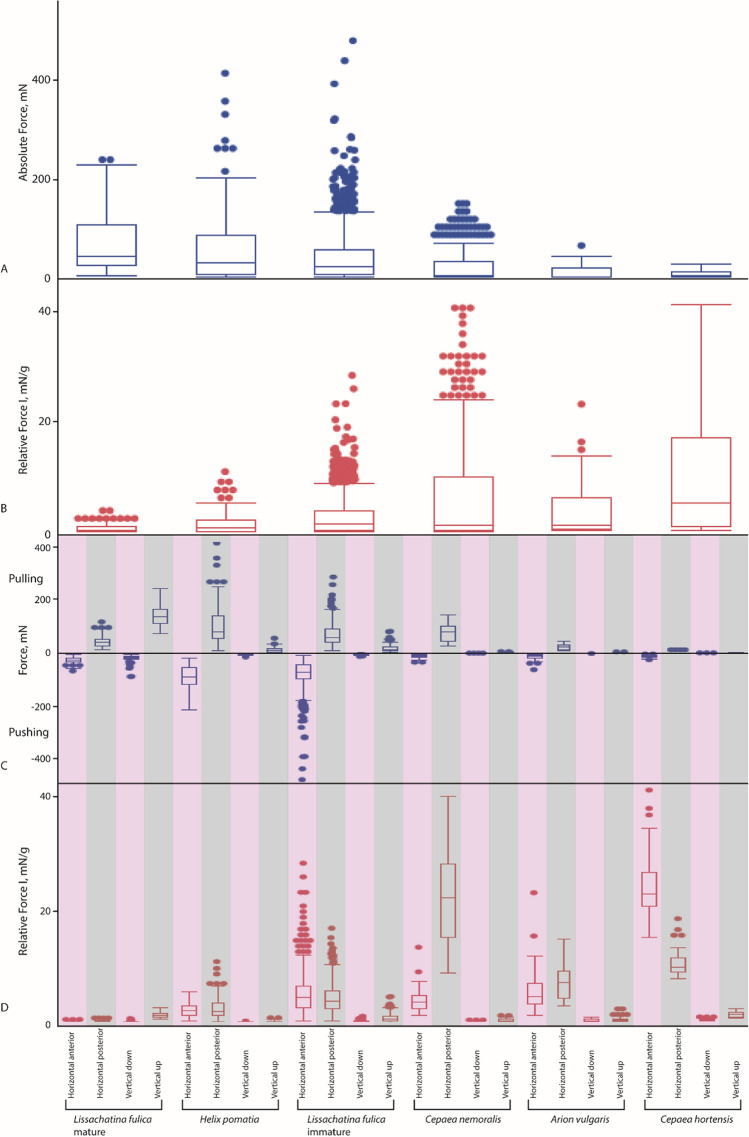
Absolute Force (blue boxplots) and Relative Force I (red boxplots) for distinct cohorts. (**A**) Absolute Force (regardless of the direction of measurement). (**B**) Relative Force I (regardless of the direction of measurement). (**C**) Absolute Force sorted to directions. (**D**) Relative Force I sorted to directions. For values of C and D see Supplementary Table 2. Pink = pushing of radula, grey = pulling of radula.

Overall, 4407 individual force peaks were evaluated. For the quantity of evaluated peaks per species, individual, and direction see Supplementary Table 1. For the Absolute Forces exerted by each body mass class see Fig. 3; for the Absolute Forces exerted by each cohort see Fig. 4 and Supplementary Table 2; for the Absolute Forces of individual gastropods see Supplementary Figs. 1, 2, and Supplementary Tables 3, 4.

### Force measurements of body mass classes

Comparing the Absolute Forces regardless of the direction between the three body mass classes of animals (Fig. 3A) we detect significant differences (*p* < 0.0001, ChiSquare: 995.6664, df: 2). Large sized individuals are capable of exerting highest forces, followed by the medium sized, and finally small sized individuals (see Table 1). When comparing the Relative Force I regardless of the direction between the three body mass classes of animals (Fig. 3B) we detect significant differences between groups (*p* < 0.0001, ChiSquare: 409.7297, df: 2). Here, small sized individuals exhibited the highest Relative Force I, followed by the medium sized, and finally large sized animals (see Table 1).

**Table 1:** For body mass classes: absolute force, mean ± SD (mN), obtained during measurements in both directions (vertical up, vertical down, horizontal posterior, horizontal anterior), and relative force I, mean ± SD (mN/g), with results from Kruskal–Wallis test, and quantity of evaluated radular force measurements (see also Fig. 4).

Body mass classes	Direction	Absolute force, mN		Kruskal–Wallis test	Relative force I, mN/g		Kruskal–Wallis test	Quantity of evaluated force measurements
		Mean	± SD		Mean	± SD		
Large sized individuals	All	73.96	62.49		0.84	0.74		1025
	Vertical up	147.35	41.35	*p* < 0.0001, ChiSquare: 744.5827, df: 3	1.69	0.54	*p* < 0.0001, ChiSquare: 750.5548, df: 3	372
	Vertical down	− 22.77	17.79		0.25	0.21		107
	Horizontal posterior	38.82	19.49		0.43	0.20		323
	Horizontal anterior	− 26.97	2.91		0.30	0.13		223
Medium sized individuals	All	41.44	49.18		2.47	2.94		2581
	Vertical up	14.72	10.40	*p* < 0.0001, ChiSquare: 1928.4320, df: 3	0.92	0.65	*p* < 0.0001, ChiSquare: 1855.5616, df: 3	988
	Vertical down	− 3.94	2.30		0.24	0.17		440
	Horizontal posterior	74.52	49.76		4.28	2.65		536
	Horizontal anterior	− 82.22	55.10		4.98	3.59		617
Small sized individuals	All	14.46	24.49		6.01	8.40		801
	Vertical up	1.48	0.72	*p* < 0.0001, ChiSquare: 636.3539, df: 3	0.85	0.51	*p* < 0.0001, ChiSquare: 625.8867, df: 3	200
	Vertical down	− 0.86	0.44		0.50	0.28		210
	Horizontal posterior	39.56	35.37		13.63	8.67		211
	Horizontal anterior	− 15.34	7.87		9.26	9.08		180

SD, standard deviation.

Absolute Forces sorted to direction (Fig. 3C) also differed significantly between body mass classes (see Table 1; for statistics see Table 2). For horizontal anterior direction, the highest forces were, however, exerted by the medium sized class, followed by the large, and finally small sized gastropods. For horizontal posterior direction, the highest forces were again exerted by the medium sized class, followed by the small sized gastropods, and finally large ones. In the direction vertical down, the large sized gastropods showed highest forces, followed by the medium, and finally small gastropods. The same is found for vertical up: large gastropods, medium, and finally small ones.

**Table 2:** For body mass classes and cohorts: results from Kruskal–Wallis test for absolute force and relative force I.

	Direction	*p*	ChiSquare	df
**Body mass classes**				
Absolut forces	Horizontal anterior	< 0.0001	3569.9093	11
	Horizontal posterior			
	Vertical down			
	Vertical up			
Relative force I	Horizontal anterior	< 0.0001	3455.7572	11
	Horizontal posterior			
	Vertical down			
	Vertical up			
**Cohorts**				
Absolut forces	Horizontal anterior	< 0.0001	3674.4431	23
	Horizontal posterior			
	Vertical down			
	Vertical up			
Relative force I	Horizontal anterior	< 0.0001	3619.3924	23
	Horizontal posterior			
	Vertical down			
	Vertical up			

When comparing the Relative Force I sorted to direction (Fig. 3D) we again detect significant differences between body mass classes (see Table 1; for statistics see Table 2). Here, for horizontal anterior direction, the highest Relative Force I was exerted by the small sized gastropods, followed by the medium, and finally large ones. For horizontal posterior direction, the highest Relative Force I was again exerted by the small, followed by the medium, and finally large sized class individuals. In the direction vertical down, the small sized gastropods showed highest Relative Force I, followed by the large, and finally medium gastropods. For vertical up direction, the large gastropods exerted highest Relative Force I, followed by the medium, and finally small ones.

### Force measurements of cohorts

When comparing Absolute Forces regardless of the direction between cohorts (Fig. 4A) we found significant differences (*p* < 0.0001, ChiSquare: 1026.4480, df: 5). Highest forces were exerted by mature *L. fulica*, followed by *H. pomatia*, immature *L. fulica*, *C. nemoralis*, *A. vulgaris*, and finally *C. hortensis* (see Supplementary Table 2). When comparing Relative Force I regardless of the direction between cohorts (Fig. 4B) we found significant differences (*p* < 0.0001, ChiSquare: 499.1690, df: 5). Highest Relative Force I was exerted by *C. hortensis*, followed by *C. nemoralis*, *A. vulgaris*, immature *L. fulica*, *H. pomatia*, and finally mature *L. fulica* (see Supplementary Table 2).

Comparing the Absolute Forces sorted to direction between cohorts we found significant differences (Fig. 4B; for values see Supplementary Table 2; for statistics see Table 2). For horizontal anterior direction, the highest forces were exerted by *H. pomatia*, followed by the immature *L. fulica*, mature *L. fulica*, *A. vulgaris*, *C. hortensis*, and finally *C. nemoralis*. For horizontal posterior direction, the highest forces were exerted by *H. pomatia*, followed by *C. nemoralis*, the immature *L. fulica*, mature *L. fulica*, *A. vulgaris*, and finally *C. hortensis*. For vertical down direction, the highest forces were exerted by mature *L. fulica*, followed by *H. pomatia*, the immature *L. fulica*, *A. vulgaris*, *C. nemoralis*, and finally *C. hortensis*. For vertical up direction, the highest forces were exerted by mature *L. fulica*, followed by the immature *L. fulica*, *H. pomatia*, *C. nemoralis*, *A. vulgaris*, and finally *C. hortensis*.

When comparing the Relative Force I sorted to direction between cohorts we found significant differences (Fig. 4C; for values see Supplementary Table 2; for statistics see Table 2). For horizontal anterior, the highest Relative Force I was exerted by *C. hortensis*, followed by *A. vulgaris*, the immature *L. fulica*, *C. nemoralis, H. pomatia*, and finally mature *L. fulica*. For horizontal posterior direction, the highest Relative Force I was exerted by *C. nemoralis,* followed by *C. hortensis, A. vulgaris*, the immature *L. fulica*, *H. pomatia*, and finally mature *L. fulica*. For vertical down direction, the highest Relative Force I was exerted by *C. hortensis*, followed by *A. vulgaris*, the immature *L. fulica*, mature *L. fulica*, *C. nemoralis*, and finally *H. pomatia*. For vertical up direction, the highest Relative Force I was exerted by mature *L. fulica*, followed by *C. hortensis*, the immature *L. fulica*, *A. vulgaris*, *C. nemoralis*, and finally *H. pomatia*.

### Masses of body, radula, buccal mass musculature, and radular sizes

Highest whole body mass (see Supplementary Table 1) was measured for *L. fulica* mature number (no.) 1, followed by *H. pomatia*, L*. fulica* immature no. 9, *C. nemoralis* no. 1, *A. vulgaris* no. 2, 4, 5, *C. nemoralis* no. 2, *A. vulgaris* no. 1, and finally *C. hortensis*.

Overall, we found that the body mass (with and without shell) relates in proportion to the masses of the whole buccal mass (wet; BRJ), the radular musculature (buccal mass musculature, wet and dry; B), and the radula and jaw (wet and dry; RJ). When individuals were heavier, they usually possessed higher muscle mass, a heavier radula and jaw (see Supplementary Fig. 8 and Supplementary Table 6). Exceptions were: *L. fulica* immature no. 9 (18 g body mass and 205.01 mg whole buccal mass) and *H. pomatia* (38 g body mass and 163.30 mg whole buccal mass), *C. nemoralis* no. 1 (3.60 g body mass and 27.20 mg whole buccal mass), and *A. vulgaris* no. 2 (3.50 g body mass and 56.66 mg whole buccal mass). Comparing mature (mature 1: 78.00 g body mass and 286.80 mg BRJ) and immature *L. fulica* (immature no. 9: 18.00 g body mass and 205.01 mg BRJ) we found that the body mass increases for the factor ~ 4 and BRJ increases for the factor 1.4.

We found that smaller gastropods are capable of exerting higher forces per whole buccal mass, radular muscle, and radula and jaw mass, dry as well as wet, (see Supplementary Fig. 9 and Supplementary Table 6). *Cepaea hortensis* exerted the highest force per radular musculature mass, followed by *C. nemoralis* no. 1, *A. vulgaris* no. 4 and 5, *C. nemoralis* no. 2, *H. pomatia*, *L. fulica* mature no. 1, *L. fulica* immature no. 9, and finally *A. vulgaris* no. 1 and 2. The same sequence was also found for force per body mass without shell. With the exception of *A. vulgaris* no. 4 and 5, exerting the highest forces per radula and jaw mass, we detected the same order for this parameter.

The radular length and width (see Supplementary Fig. 8 and Supplementary Table 5) do not consistently correlate with body mass, the highest radular width was measured for *L. fulica* mature no. 1, followed by *A. vulgaris* no. 5, *L. fulica* immature no. 9, *H. pomatia*, *A. vulgaris* no. 4, *A. vulgaris* no. 1, *A. vulgaris* no. 2, *C. nemoralis* no. 2, *C. nemoralis* no. 1, and finally *C. hortensis*. Both gastropods with the largest width possessed shorter radulae than all other specimens. *L. fulica* immature no. 9 possessed the largest radular area, followed by *H. pomatia*, *L. fulica* mature no. 1, *A. vulgaris* no. 4, *A. vulgaris* no. 2, *A. vulgaris* no. 5, *C. nemoralis* no. 1, *A. vulgaris* no. 1, *C. nemoralis* no. 2, and finally *C. hortensis*. The radular area again did not consistently correlate with the whole body mass.

### Force quotients

**Figure 5:**
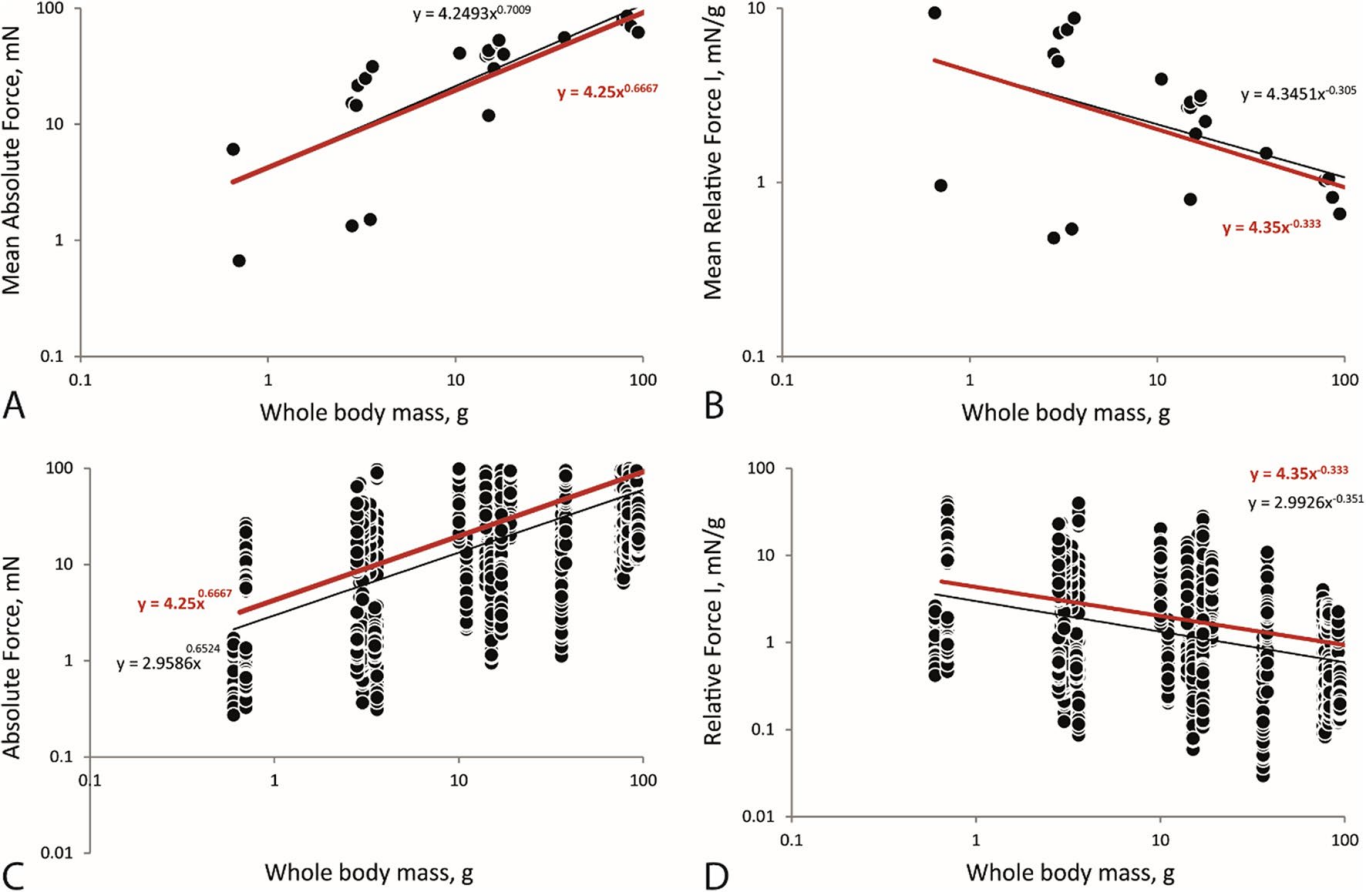
Linear regression, displayed on logarithmic axes, with trend lines (black = real trend line; red = calculated trend line for the factors 4.25 and 4.35), regardless of the direction. (**A**) Mean Absolute Force *versus* mean whole body mass. A radular force quotient, AFQ = mean Absolute Force/Bodymass^0.67^, of 4.25 was determined. (**B**) Mean Relative Force I *versus* mean whole body mass. A Relative Force Quotient, RFQ = mean Relative Force I/Bodymass^−0.33^, of 4.35 was determined. (**C**) Variance of Absolute Force *versus* mean whole body mass. (**D**) Variance of Relative Force I *versus* mean whole body mass. For C and D a force quotient of 3.00 was determined.

**Figure 6:**
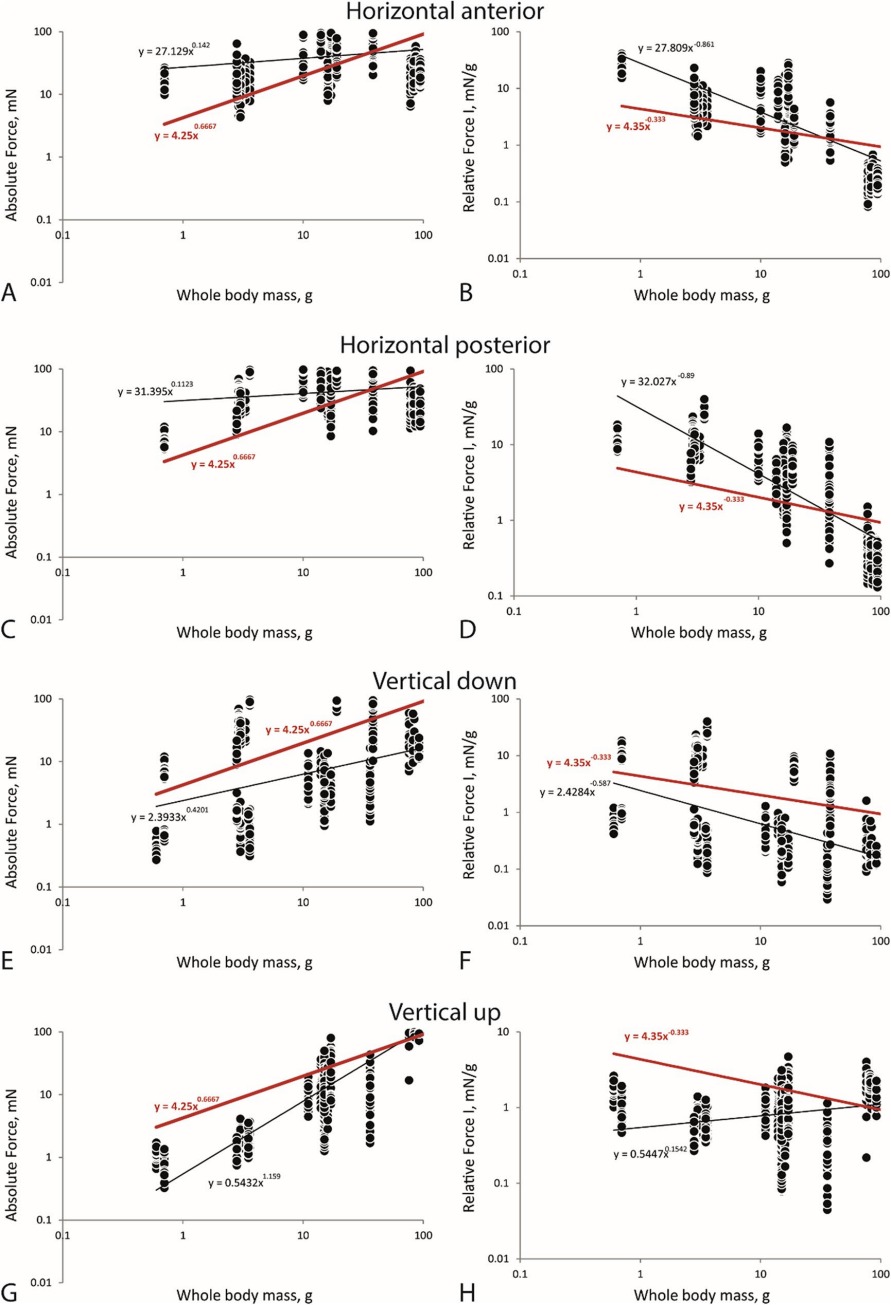
Linear regression, displayed on logarithmic axes, with trend lines (black = real trend line; red = calculated trend line for the factors 4.25 and 4.35), for each direction (**A**–**B**: horizontal anterior, **C**–**D**: horizontal posterior, **E**–**F**: vertical down, **G**–**H**: vertical up). Left side (**A**, **C**, **E**, **G**). Variance of Absolute Force *versus* mean whole body mass. Right side (**B**, **D**, **F**, **H**). Variance of Relative Force I *versus* mean whole body mass.

We found that the stylommatophoran species perfectly follow the predictions for scaling of force and body mass as the mean Absolute radular Force (regardless of the direction) scales to body mass^0.67^ with the quotient 4.25 and the mean Relative Force I (regardless of the direction) scales to body mass^−0.33^ with the quotient 4.35 (Fig. 5A,B). When the variance of the Absolute Force (regardless of the direction) is scaled to body mass^0.67^ and the variance of the Relative Force I (regardless of the direction) is scaled to body mass^−0.33^ we receive quotients of 3 (Fig. 5C,D). When the forces are sorted to directions the picture is rather puzzling: for horizontal anterior a quotient of 27 (Fig. 6A,B), for horizontal posterior a quotient of 31–32 (Fig. 6C,D), for vertical down a quotient of 2.4 (Fig. 6E,F), and for vertical up a quotient of 0.54 (Fig. 6G,H) is calculated.

## Discussion

Force output is often referred to as proportional to muscle mass^0.67^, the muscle cross-sectional area^95,96^, or to body mass^0.67^^97^ whereas the forces, corrected for body mass (force/mass), are referred to be proportional to body mass^−0.33^^97^. The stylommatophoran species examined here, follow the predictions for scaling of force and body mass for mean values. Following previous studies^22,98^ we here experimentally determined a Radular Force Quotient AFQ, AFQ = mean Absolute Force/Bodymass^0.67^, of 4.25 and a Relative Force Quotient RFQ, RFQ = mean Relative Force I/Bodymass^−0.33^ of 4.35 which can be further used for predictions of forces in Gastropoda. However, when the forces are sorted to directions the picture becomes puzzling, indicating the need for further studies.

The here measured Absolute feeding Forces for *Helix pomatia* are in a similar range as to those documented by^87^ for *Cornu aspersum*, both gastropod groups have comparable body mass. We detected that gastropods with a higher body mass and a larger body size were capable of exerting higher radular forces, which is not surprising. However, the relationship between forces generated by the feeding organ and body size is well documented for vertebrates, but not for molluscs. When forces are corrected for body size or mass, ecological adaptations related with this parameter usually became more pronounced (e.g.^4,7,16,99,100^; for hypotheses about body size related evolution of bite force, see also^101,102^). When determining the radular force per body mass (termed Relative Force I), we see that smaller gastropods were capable of exerting the highest forces, followed by the medium, and finally large ones, similar to e.g. carnivorans in placental mammals^7^. This is also not surprising, because of different scaling of body mass and cross-sectional area of muscles^103,104^. Usually, the sampled gastropod specimens exhibiting a higher body mass also possess a proportionally higher mass of buccal mass (correlation between buccal mass size and gastropod body size was also previously described by^51^). However, the immature and adult *Lissachatina fulica,* exhibiting strong differences in body mass and size, but having almost similar buccal mass sizes and masses, are an exception to this rule. In most experiments mature *L. fulica* exerted higher radular forces than the immature gastropods, but some measurements (outliers) revealed that immature *L. fulica* are also capable of generating the same feeding forces as the mature ones. This could be an indication that the ingesta type does not change during ontogeny; however, this aspect awaits further investigation. But our analyses of Relative Force I (Fig. 4) reveal distinct radular force patterns for cohorts, both *Cepaea* species have a wide range of exerted forces, followed by *Arion*, *Lissachatina* immature, and finally, with the smallest range, *Lissachatina* mature. This could be an indication that species have species-specific radular forces. *Cepaea* potentially feeds naturally on a broader food spectrum whereas *Lissachatina* is more restricted, but this also awaits further investigations. *Arion* was fed with fresh strawberries, since it did not want to feed on carrots. This could have influenced the forces generated; potentially *Arion* is capable of exerting higher forces. It should be additionally stated that here the whole body masses were related to the radular forces generated. But, as we experimented with four snail species and one slug species (*Arion vulgaris*), whole body masses are not directly comparable, because the mass of the shell could cause artefacts. In future studies the body mass without shell should be determined persistently to detect a potentially more accurate relationship or even correlation.

In past studies, it has been shown that the forces generated usually correlate with the muscle mass, muscle size, or muscle diameter (for vertebrates: e.g.^8,16,105,106^; for invertebrates:^107^). This is congruent to the here observed patterns in gastropods. However, for precise interpretation of the relationship between buccal mass size and the mass of the radular muscles (buccal mass musculature) knowledge about the functional role of each feeding muscle must be available, which is not the case for the taxa studied here. There are some detailed studies analyzing the in vivo buccal mass movement and buccal mass muscle function in *Aplysia*^89,90,92,108^. But since *Aplysia* belongs to the Opisthobranchia and is not closely related to the taxa involved in this experiment the function of the buccal mass muscles for these stylommatophoran taxa cannot be assigned yet. However, to deeply understand the relationship between measured radular forces involved in specific radular motions, the function of each muscle, their work in concert, as well as the muscle and fiber size, length, or diameter need to be investigated. As there are many different parameters that relate with feeding force (for vertebrates: e.g. the skull geometry and size:^10,17,99,109–111^; or muscular development:^105^), it is very difficult to produce reliable models for molluscs due to the lack of solid experimental data. Additionally, studies on invertebrates reveal that muscle stress varies considerably depending on the muscle^112–114^. This could also be the case for molluscs as the forces exerted per radular muscular mass differ extremely between the analyzed stylommatophoran individuals (see Supplementary Fig. 9 and Supplementary Table 6). This again shows that pure anatomy-based studies on the muscle systems of invertebrates do not necessarily provide data on physiology (see also^24^). Additionally, the area of the radula used for foraging could influence results, but the working area could so far only be determined by involving sandpapers of different roughness^115^. Since sandpaper is a rather flat surface in comparison to a slice of carrot we cannot directly translate our past findings to the experiments here.

Our results clearly indicate that larger and heavier animals exerted higher forces in the vertical directions, whereas medium and small individuals exerted higher forces in horizontal directions. This is additionally supported by the analyses of the video footage showing that larger animals rather pull with radula and jaw, whereas smaller individuals use their radular often like a saw in anterior–posterior direction when approaching the food item (see Supplementary Video 1 and 2). This shift in feeding behavior seems to depend on the ingesta size in relation to the mouth opening size. Smaller and medium sized gastropods—even though capable of embracing the whole item with the lip—seem to prefer this alternative feeding pattern. An ingesta-depending shift in feeding pattern, i.e. dynamics of swallowing, had been documented for other gastropod taxa when altering parameters of the ingesta^108^, e.g. its hardness^116^, its load and width^117^, or its size^118–120^. Additionally, gastropods of different sizes have been found to feed on different ingesta types, possibly correlating with ontogenetic size changes of the mouthparts^121^.

As already stated above, heavier gastropods were usually capable of exerting higher radular forces than smaller ones (except for immature and adult *L. fulica*). This indicates that larger gastropods might forage on a broader spectrum of ingesta, as it had been previously described for turtles^109^. However, the smaller individuals were able to exert higher forces per body mass and additionally show a distinct radular motion pattern resulting in a distinct feeding pattern. All experiments resulted in the consumption of the ingesta offered, but smaller gastropods invested more time. This effect had also been observed for lizards^122^ and should be further investigated for Mollusca. Thus, we can conclude that in studies of feeding forces of gastropods with a similar radular type, teeth, and radular motion, adaptations to ingesta might only been detected by studying feeding efficiency and time invested. We hope that in the future more biomechanical, physiological and functional morphological studies will approach the topic of trophic specialization in molluscs via feeding force experiments on species possessing distinct radular morphologies and feeding on more types of ingesta.

## Supplementary Information

Supplementary Information 1.

Supplementary Information 2.

Supplementary Information 3.

## Data Availability

The datasets generated and analysed during the current study (the force measurements) are available from the corresponding author on reasonable request; all other datasets are included in this published article (and its Supplementary Information files).
