# Supplementary material for: Radular force performance of stylommatophoran gastropods (Mollusca) with distinct body masses
Source: Sci Rep. 2021 May 18;11:10560. doi: 10.1038/s41598-021-89892-z (PMC8131350; doi:10.1038/s41598-021-89892-z)
Supplement: Supplementary file 1 — Supplementary Information 1. [file 41598_2021_89892_MOESM1_ESM.pdf]

Supplementary

Radular force performance of stylommatophoran gastropods (Mollusca) with distinct body masses

WENCKE KRINGS[1,3*], CHARLOTTE NEUMANN[1], MARCO T. NEIBER[2], ALEXANDER KOVALEV[3], STANISLAV N. GORB[3]

[1] Department of Mammalogy and Palaeoanthropology, Center of Natural History (CeNak), Universität Hamburg, Martin-Luther-King-Platz 3, 20146 Hamburg, Germany

[2] Department of Animal Diversity, Center of Natural History (CeNak), Universität Hamburg, Martin-Luther-King-Platz 3, 20146 Hamburg, Germany

[3] Department of Functional Morphology and Biomechanics, Zoological Institute, Christian-Albrechts-Universität zu Kiel, Am Botanischen Garten 9, 24118 Kiel, Germany

*corresponding author: wencke.krings@uni-hamburg.de

Supplementary Video 1. Feeding sequence of mature *Lissachatina fulica*.

Supplementary Video 1.mp4

Supplementary Video 2. Feeding sequence of immature *Lissachatina fulica* involving more feeding motions along the horizontal axis.

Supplementary Video 2.mp4

Supplementary Table 1. Quantity of radular force measurements per individual performed in directions (vertical down, vertical up, horizontal anterior, horizontal posterior) and body mass (with shell and without shell; g) of individual animals studied. SD, standard deviation.

Species	Individual	Direction of force				Body mass with shell, g		Body mass, without shell, g
		Vertical down	Horizontal anterior	Vertical up	Horizontal posterior	Mean	± SD	
Lissachatina fulica	Mature 1	20	42	92	80	78.00	0.99	-
	Mature 2	17	40	60	62	86.00	0.99	-
	Mature 3	11	22	60	41	82.00	0.87	-
	Mature 4	11	40	60	40	94.00	0.99	-
	Mature 5	48	79	100	100	102.00	1.13	-
	**Mature all**	**107**	**223**	**372**	**323**			
	Immature 1	22	115	201	58	15.00	0.50	-
	Immature 2	43	0	160	0	15.00	0.50	-
	Immature 3	0	78	120	70	17.00	-	-
	Immature 4	81	90	94	90	15.00	0.49	-
	Immature 5	63	54	143	60	16.00	0.50	-
	Immature 6	51	90	90	58	17.00	0.45	-
	Immature 7	40	40	40	40	14.50	0.50	-
	Immature 8	40	40	40	40	10.50	0.50	-
	Immature 9	40	40	40	40	18.00	1.00	14.10
	**Immature all**	**380**	**547**	**928**	**456**			
Helix pomatia	Mature 1	60	70	60	80	38.00	0.99	31.70
Cepaea nemoralis	Mature 1	40	35	40	41	3.60	-	3.10
	Mature 2	40	40	40	40	2.95	0.05	2.25
	**Mature all**	**80**	**75**	**80**	**81**			
Cepaea hortensis	Mature 1	**40**	**40**	**40**	**40**	0.65	0.05	0.33
Arion vulgaris	Mature 1	30	0	20	0	-	-	2.80
	Mature 2	30	0	30	0	-	-	3.50
	Mature 3	30	0	30	0	-	-	0.70
	Mature 4	0	18	0	30	-	-	3.30
	Mature 5	0	17	0	30	-	-	3.00
	Mature 6	0	30	0	30	-	-	2.80
	**Mature all**	**90**	**65**	**80**	**90**			

Supplementary Table 2. For cohorts: Absolute Force, mean ± SD (mN), obtained during measurements in both directions (vertical up, vertical down, horizontal posterior, horizontal anterior), Relative Force I, mean ± SD (mN/g), with results from Kruskal-Wallis test, and quantity of evaluated radular force measurements (see also Fig. 4). SD, standard deviation.

Cohort	Direction	Absolute Force, mN		Kruskal-Wallis Test	Relative Force I, mN/ g		Kruskal-Wallis Test	Quantity of evaluated force measurements
		Mean	± SD		Mean	± SD		
**Mature *Lissachatina fulica***	All	73.96	62.49		0.84	0.74		1025
	Vertical up	147.35	41.35	p<0.0001, ChiSquare: 744.5827, df: 3	1.69	0.54	p<0.0001, ChiSquare: 750.5548, df: 3	372
	Vertical down	-22.77	17.79		-0.25	0.21		107
	Horizontal posterior	38.82	19.49		0.43	0.20		323
	Horizontal anterior	-26.97	12.24		-0.30	0.13		223
Helix pomatia	All	55.98	65.25		1.47	1.72		270
	Vertical up	10.77	9.16	p<0.0001, ChiSquare: 196.1349, df: 3	0.28	0.24	p<0.0001, ChiSquare: 196.1349, df: 3	60
	Vertical down	-4.60	2.91		-0.12	0.08		60
	Horizontal posterior	100.88	81.25		2.65	2.14		80
	Horizontal anterior	-87.47	39.28		-2.30	1.03		70
**Immature *Lissachatina fulica***	All	39.73	46.67		2.59	3.03		2311
	Vertical up	14.97	10.42	p<0.0001, ChiSquare: 1725.9192, df: 3	0.96	0.64	p<0.0001, ChiSquare: 1715.4599, df: 3	928
	Vertical down	-3.84	2.18		-0.26	0.17		380
	Horizontal posterior	69.90	40.27		4.56	2.63		456
	Horizontal anterior	-81.54	56.80		-5.33	3.66		547
Cepaea nemoralis	All	23.01	35.08		6.85	9.99		316
	Vertical up	2.00	0.67	p<0.0001, ChiSquare: 292.5987, df: 3	0.63	0.25	p<0.0001, ChiSquare: 292.4164, df: 3	80
	Vertical down	-0.71	0.31		-0.22	0.10		80
	Horizontal posterior	74.64	32.90		22.09	8.01		81
	Horizontal anterior	-13.44	6.62		-4.08	1.94		75
Arion vulgaris	All	10.25	11.79		3.52	3.72		325
	Vertical up	1.17	0.60	p<0.0001, ChiSquare: 245.8172, df: 3	0.70	0.38	p<0.0001, ChiSquare: 245.4777, df: 3	80
	Vertical down	-1.16	0.44		-0.64	0.22		90
	Horizontal posterior	22.46	9.29		7.28	2.76		90
	Horizontal anterior	-17.13	10.27		-5.77	3.57		65
Cepaea hortensis	All	6.10	6.60		9.43	10.17		160
	Vertical up	1.04	0.28	p<0.0001, ChiSquare: 147.7844, df: 3	1.60	0.43	p<0.0001, ChiSquare: 147.7844, df: 3	40
	Vertical down	-0.47	0.11		-0.72	0.17		40
	Horizontal posterior	7.00	1.54		10.77	2.37		40
	Horizontal anterior	-16.01	3.93		-24.63	6.04		40

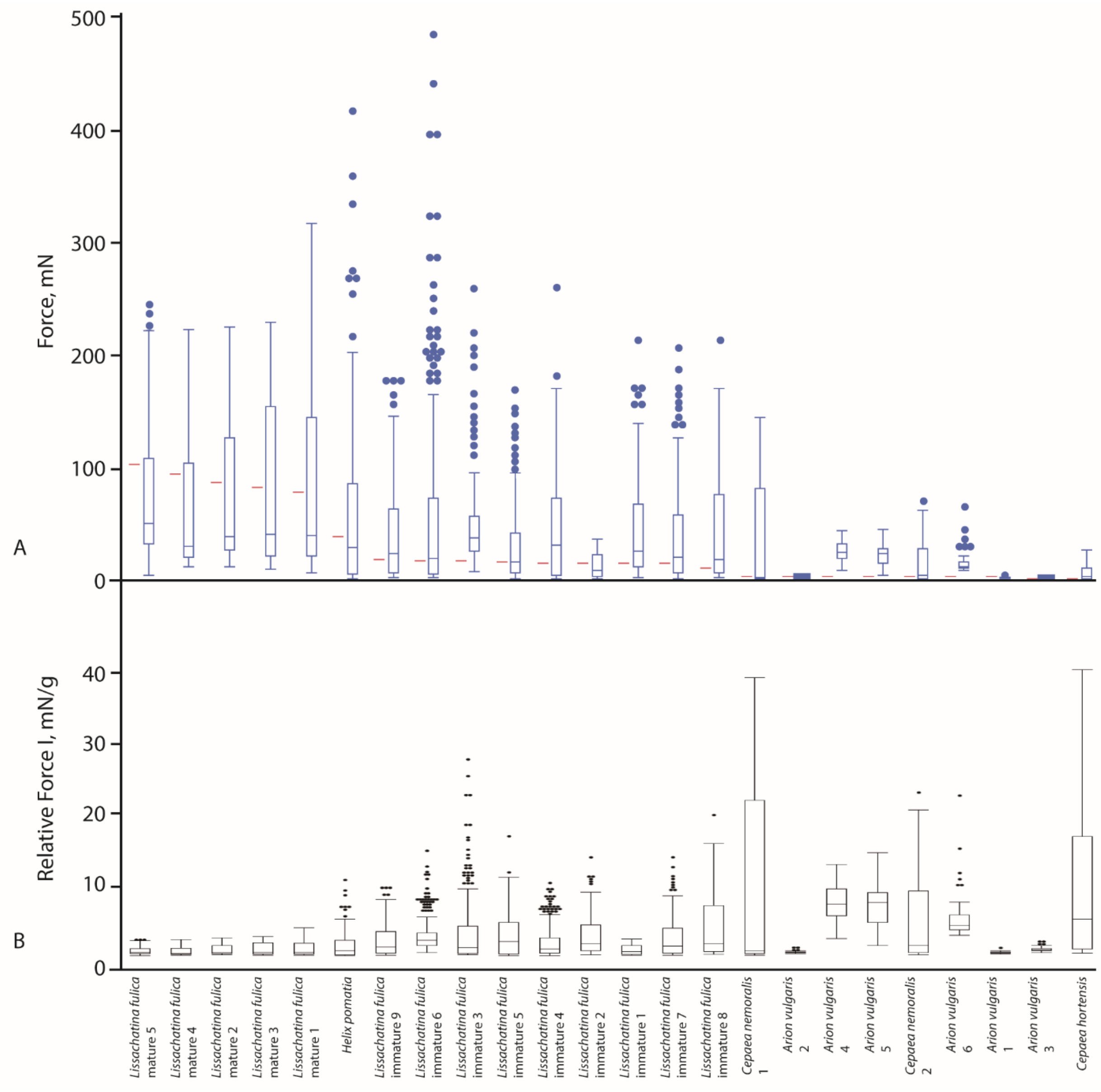

Supplementary Figure 1. Absolute Force (blue boxplots) and Relative Force I (black boxplots) for individual gastropods studied (sorted by descending mean body mass = red points in the above graph). A. Absolute Force (regardless of the direction of measurement). B. Relative Force I (regardless of the direction of measurement). Significant differences were detected between individual gastropods (for Absolute Force: p <0.0001, F-ratio: 39.55, df: 23; for Relative Force I: p <0.0001, F-ratio: 61.39, df: 23). For original values see Supplementary Tab. 3.

Supplementary Table 3. Absolute Force (regardless of the direction of measurement), mean ± SD (mN), Relative Force I, mean ± SD (mN/g) for each individual gastropod (see also Supplementary Fig. 1), and quantity of evaluated force measurements. SD, standard deviation.

Species	Individual	Direction	Absolute Force, mN		Relative Force I, mN/g		Quantity of evaluated force measurements
			Mean	± SD	Mean	± SD	
Lissachatina fulica	Mature 1	All	80.42	72.33	1.03	0.93	234
	Mature 2	All	70.42	60.33	0.82	0.70	179
	Mature 3	All	85.93	70.64	1.05	0.86	134
	Mature 4	All	62.18	52.49	0.66	0.56	151
	Mature 5	All	71.80	55.42	0.70	0.54	327
	Immature 1	All	40.40	36.66	2.69	2.44	396
	Immature 2	All	11.95	9.54	0.80	0.64	203
	Immature 3	All	50.65	41.65	2.98	2.45	268
	Immature 4	All	43.39	44.17	2.89	2.94	355
	Immature 5	All	30.38	35.59	1.90	2.22	320
	Immature 6	All	53.33	77.20	3.14	4.54	289
	Immature 7	All	39.15	42.98	2.70	2.96	160
	Immature 8	All	41.14	45.65	3.92	4.35	160
	Immature 9	All	40.31	43.97	2.24	2.44	160
Helix pomatia	Mature 1	All	55.98	65.25	1.47	1.71	270
Cepaea nemoralis	Mature 1	All	31.60	44.67	8.78	12.41	156
	Mature 2	All	14.63	18.72	4.96	6.35	160
Cepaea hortensis	Mature 1	All	6.13	0.41	9.43	10.17	50
Arion vulgaris	Mature 1	All	1.34	0.43	0.48	0.15	50
	Mature 2	All	1.52	0.22	0.54	0.15	60
	Mature 3	All	0.67	9.26	0.96	0.31	60
	Mature 4	All	24.93	8.99	7.56	2.81	48
	Mature 5	All	21.73	9.31	7.24	3.00	47
	Mature 6	All	15.28	0.41	5.46	3.33	60

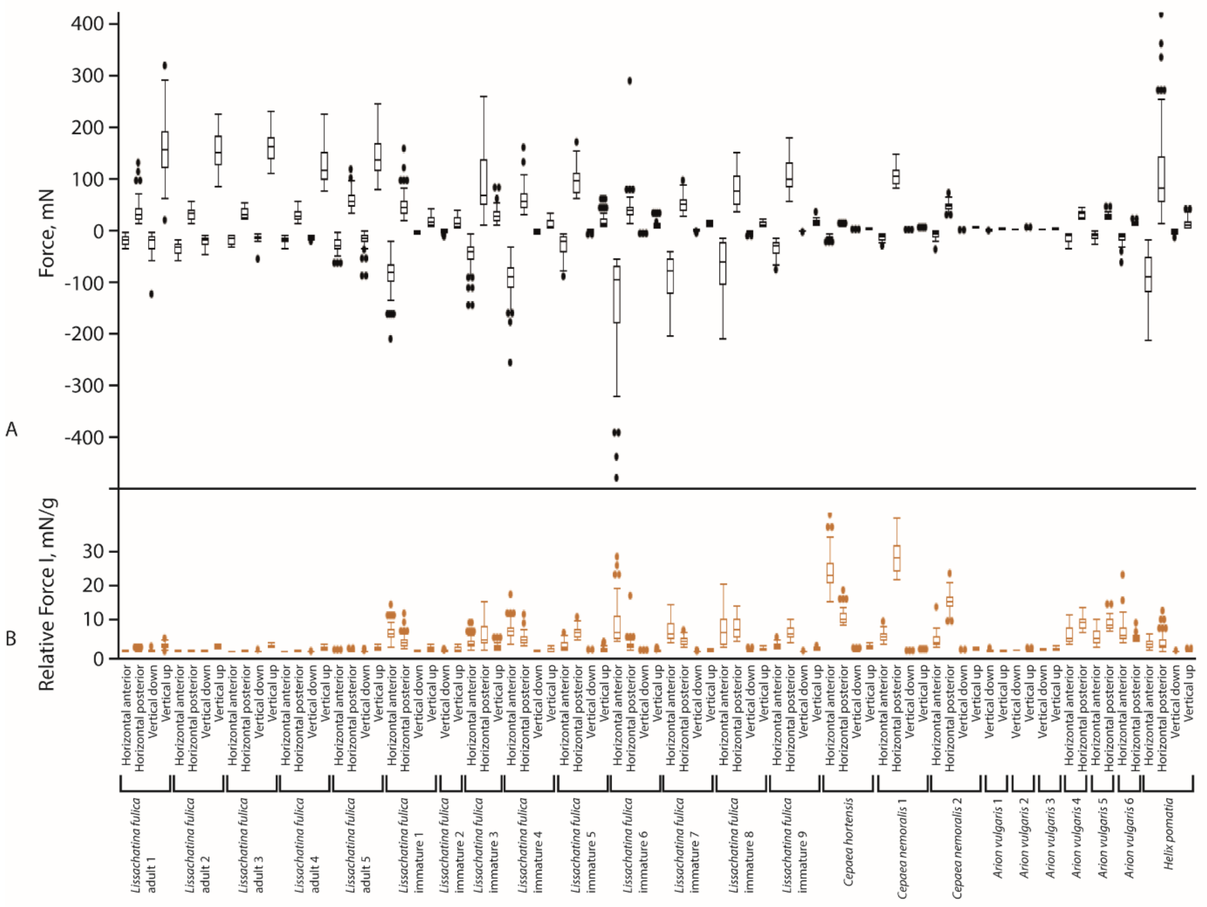

Supplementary Figure 2. Absolute Force (black boxplots) and Relative Force I (orange boxplots) for individual gastropods (sorted by descending mean body mass). A. Absolute Force sorted to directions. B. Relative Force I sorted to directions. Significant differences were detected between individual gastropods (for Absolute Force: $p<0.0001$, ChiSquare: 3914.1051, df: 80; for Relative Force I: $p<0.0001$, ChiSquare: 3868.2537, df: 80). For values see Supplementary Tab. 4.

Supplementary Table 4. Absolute Force values sorted to directions, mean ± SD (mN), Relative Force I, mean ± SD (mN/g), for each individual gastropod (see also Supplementary Fig. 2), and quantity of evaluated force measurements. SD, standard deviation.

Species	Individual	Direction	Absolute Force, mN		Relative Force I, mN/g		Quantity of evaluated force measurements
			Mean	± SD	Mean	± SD	
Lissachatina fulica	Mature 1	Horizontal anterior	-20.76	7.95	0.27	0.10	42
		Horizontal posterior	34.23	21.22	0.44	0.27	80
		Vertical down	-29.03	26.45	0.37	0.34	20
		Vertical up	158.10	49.88	2.04	0.64	92
	Mature 2	Horizontal anterior	-37.03	10.64	0.43	0.12	40
		Horizontal posterior	28.74	10.64	0.33	0.12	62
		Vertical down	-23.22	8.50	0.27	0.10	17
		Vertical up	149.13	35.30	1.73	0.41	60
	Mature 3	Horizontal anterior	-20.02	7.70	0.24	0.09	22
		Horizontal posterior	30.04	9.09	0.37	0.11	41
		Vertical down	-18.91	13.43	0.23	0.16	11
		Vertical up	160.59	29.07	1.96	0.35	60
	Mature 4	Horizontal anterior	-20.90	5.53	0.22	0.06	40
		Horizontal posterior	28.04	10.34	0.30	0.11	40
		Vertical down	-15.70	3.63	0.17	0.04	11
		Vertical up	120.99	32.33	1.29	0.34	60
	Mature 5	Horizontal anterior	-30.20	13.77	0.30	0.13	79
		Horizontal posterior	56.67	15.79	0.56	0.15	100
		Vertical down	-22.50	18.26	0.22	0.18	48
		Vertical up	143.45	39.76	1.41	0.39	100
	Immature 1	Horizontal anterior	-85.77	29.24	5.72	1.95	115
		Horizontal posterior	47.38	25.44	3.16	1.70	58
		Vertical down	-5.89	2.17	0.39	0.14	22
		Vertical up	16.20	8.02	1.08	0.53	201
	Immature 2	Vertical down	-4.13	3.13	0.28	0.21	43
		Vertical up	14.05	9.59	0.94	0.64	160
	Immature 3	Horizontal anterior	-50.13	25.57	2.95	1.50	78

		Horizontal posterior	90.23	56.61	5.31	3.33	70
		Vertical up	27.91	12.98	1.64	0.76	120
	Immature 4	Horizontal anterior	-98.26	35.18	6.55	2.35	90
		Horizontal posterior	59.70	21.95	3.98	1.46	90
		Vertical down	-3.86	1.80	0.26	0.12	81
		Vertical up	9.31	8.52	0.62	0.57	94
	Immature 5	Horizontal anterior	-31.61	20.18	1.98	1.26	54
		Horizontal posterior	94.54	26.65	5.91	1.67	60
		Vertical down	-3.42	1.83	0.21	0.11	63
		Vertical up	14.88	9.56	0.93	0.60	143
	Immature 6	Horizontal anterior	-135.11	89.71	7.95	5.28	90
		Horizontal posterior	41.44	35.76	2.44	2.10	58
		Vertical down	-3.85	1.80	0.23	0.11	51
		Vertical up	7.26	3.34	0.43	0.20	90
	Immature 7	Horizontal anterior	-94.59	42.69	6.52	2.94	40
		Horizontal posterior	48.74	15.95	3.36	1.10	40
		Vertical down	-2.91	1.54	0.20	0.11	40
		Vertical up	10.34	2.65	0.71	0.18	40
	Immature 8	Horizontal anterior	-71.05	50.55	6.77	4.81	40
		Horizontal posterior	79.10	33.90	7.53	3.23	40
		Vertical down	-4.74	2.94	0.45	0.28	40
		Vertical up	9.65	3.49	0.92	0.33	40
	Immature 9	Horizontal anterior	-37.37	14.74	2.08	0.82	40
		Horizontal posterior	106.19	32.88	5.90	1.83	40
		Vertical down	-3.05	0.84	0.17	0.05	40
		Vertical up	14.63	4.69	0.81	0.26	40
Helix pomatia	Mature 1	Horizontal anterior	-87.47	39.28	2.30	1.03	70
		Horizontal posterior	100.88	81.25	2.65	2.14	80
		Vertical down	-4.60	2.91	0.12	0.08	60
		Vertical up	10.77	9.16	0.28	0.24	60
Cepaea nemoralis	Mature 1	Horizontal anterior	-16.72	5.23	4.64	1.45	35

		Horizontal posterior	103.67	18.38	28.80	5.11	41
		Vertical down	-0.66	0.27	0.18	0.08	40
		Vertical up	1.70	0.47	0.47	0.13	40
	Mature 2	Horizontal anterior	-10.58	6.42	3.59	2.18	40
		Horizontal posterior	44.88	8.89	15.22	3.01	40
		Vertical down	-0.76	0.33	0.26	0.11	40
		Vertical up	2.31	0.70	0.78	0.24	40
Cepaea hortensis	Mature 1	Horizontal anterior	-16.01	3.93	24.63	6.04	40
		Horizontal posterior	7.00	1.54	10.77	2.37	40
		Vertical down	-0.47	0.11	0.72	0.17	40
		Vertical up	1.04	0.28	1.60	0.43	40
Arion vulgaris	Mature 1	Vertical down	-1.44	0.39	0.51	0.14	30
		Vertical up	1.18	0.39	0.42	0.14	20
	Mature 2	Vertical down	-1.39	0.12	0.50	0.04	30
		Vertical up	1.64	0.57	0.59	0.20	30
	Mature 3	Vertical down	-0.64	0.09	0.92	0.12	30
		Vertical up	0.70	0.29	1.00	0.42	30
	Mature 4	Horizontal anterior	-17.63	9.16	5.34	2.78	18
		Horizontal posterior	29.32	6.06	8.88	1.84	30
	Mature 5	Horizontal anterior	-13.59	7.33	4.53	2.44	17
		Horizontal posterior	26.34	6.14	8.78	2.05	30
	Mature 6	Horizontal anterior	-18.84	11.97	6.73	4.28	30
		Horizontal posterior	11.72	2.62	4.18	0.94	30

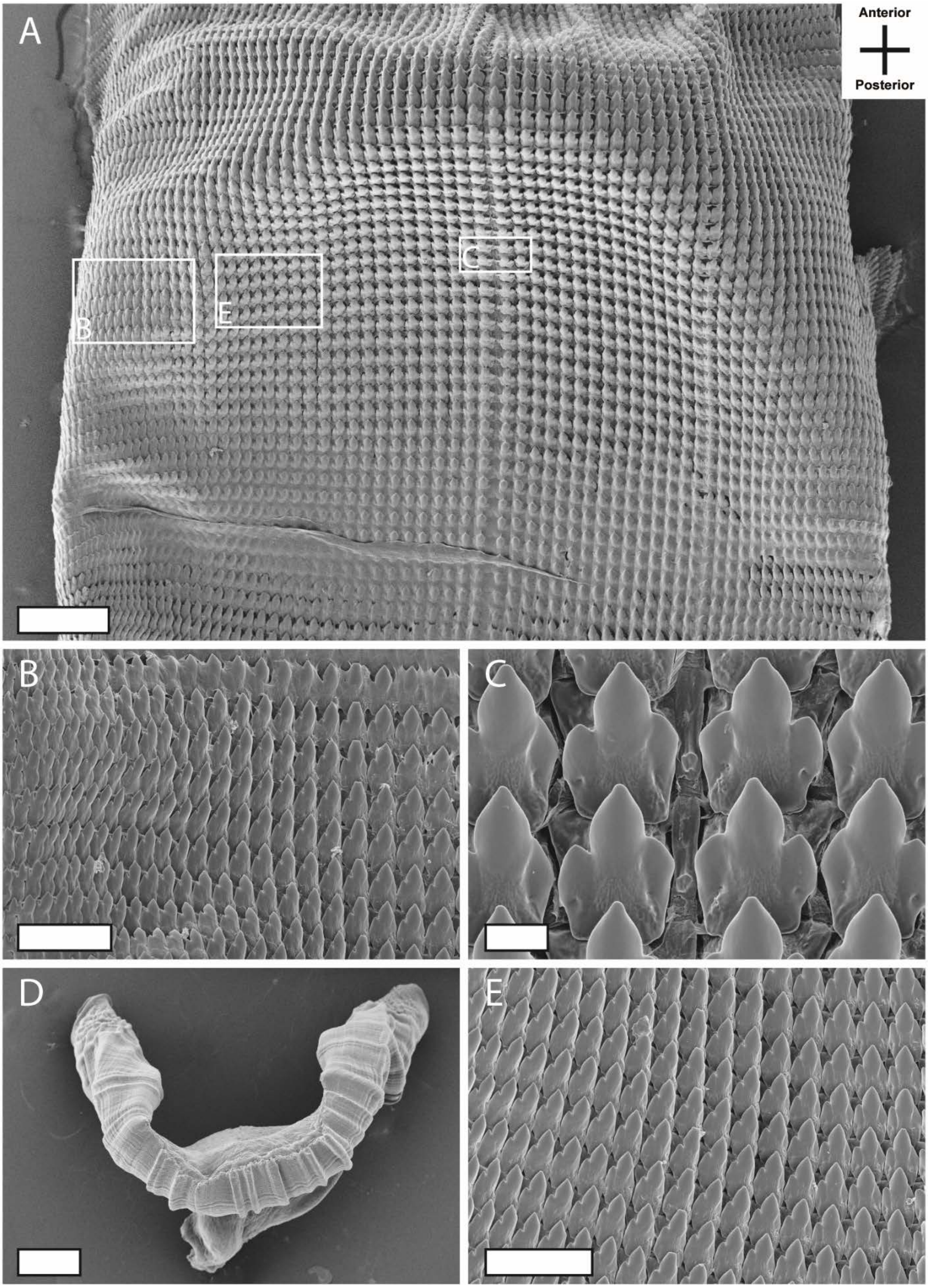

Supplementary Figure 3. SEM images of feeding organs of *Lissachatina fulica* (immature 9). A. Radula. B. Marginal teeth. C. Central and lateral teeth. D. Jaw. E. Marginal teeth. Scale bars: A, D = 400 µm, B, E = 200 µm, C = 40 µm.

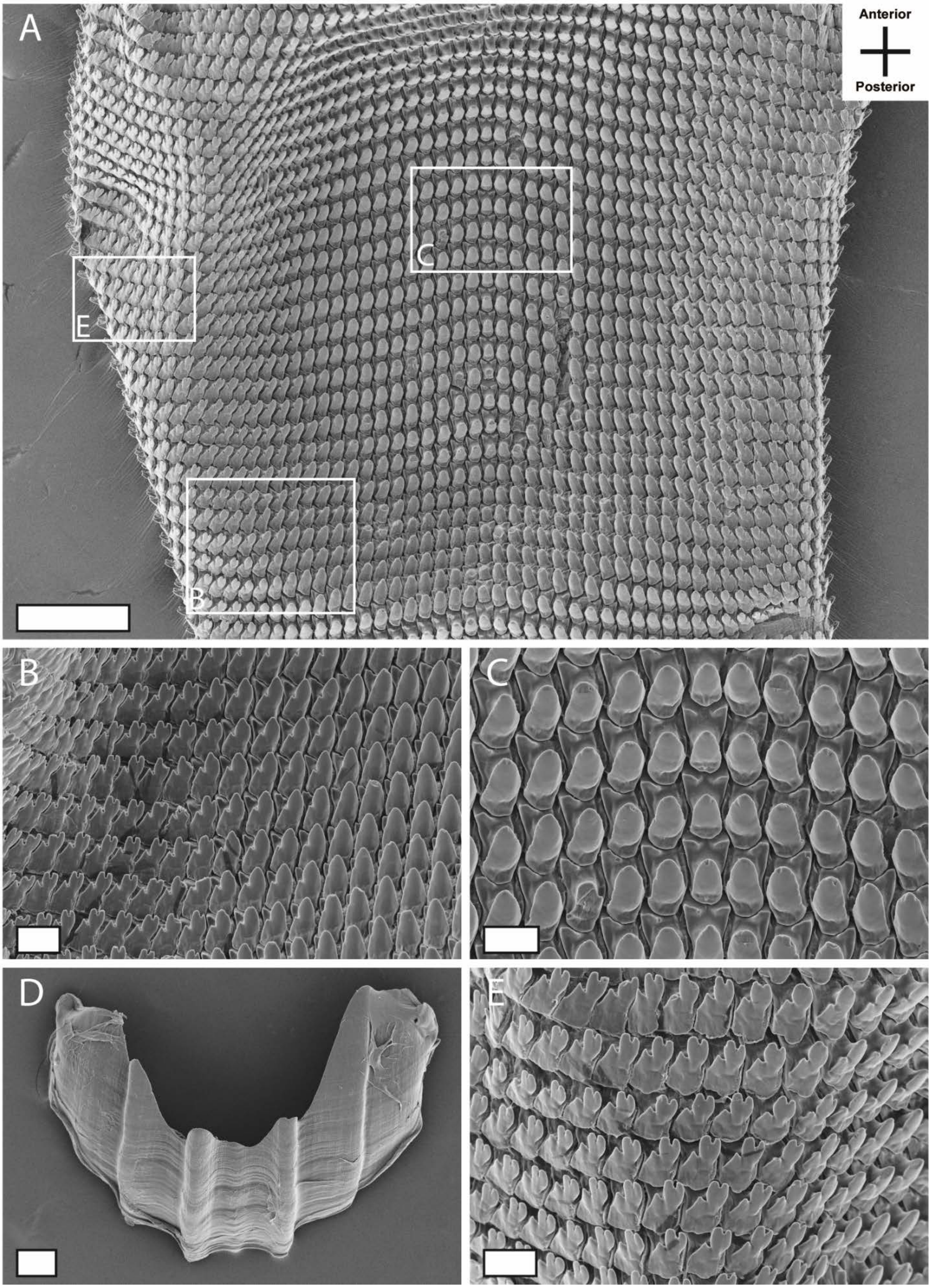

Supplementary Figure 4. SEM images of feeding organs of *Cepaea nemoralis* (specimen 2). A. Radula. B. Marginal teeth. C. Central and lateral teeth. D. Jaw. E. Marginal teeth. Scale bars: A, D = 200 µm, B, C, E = 40 µm.

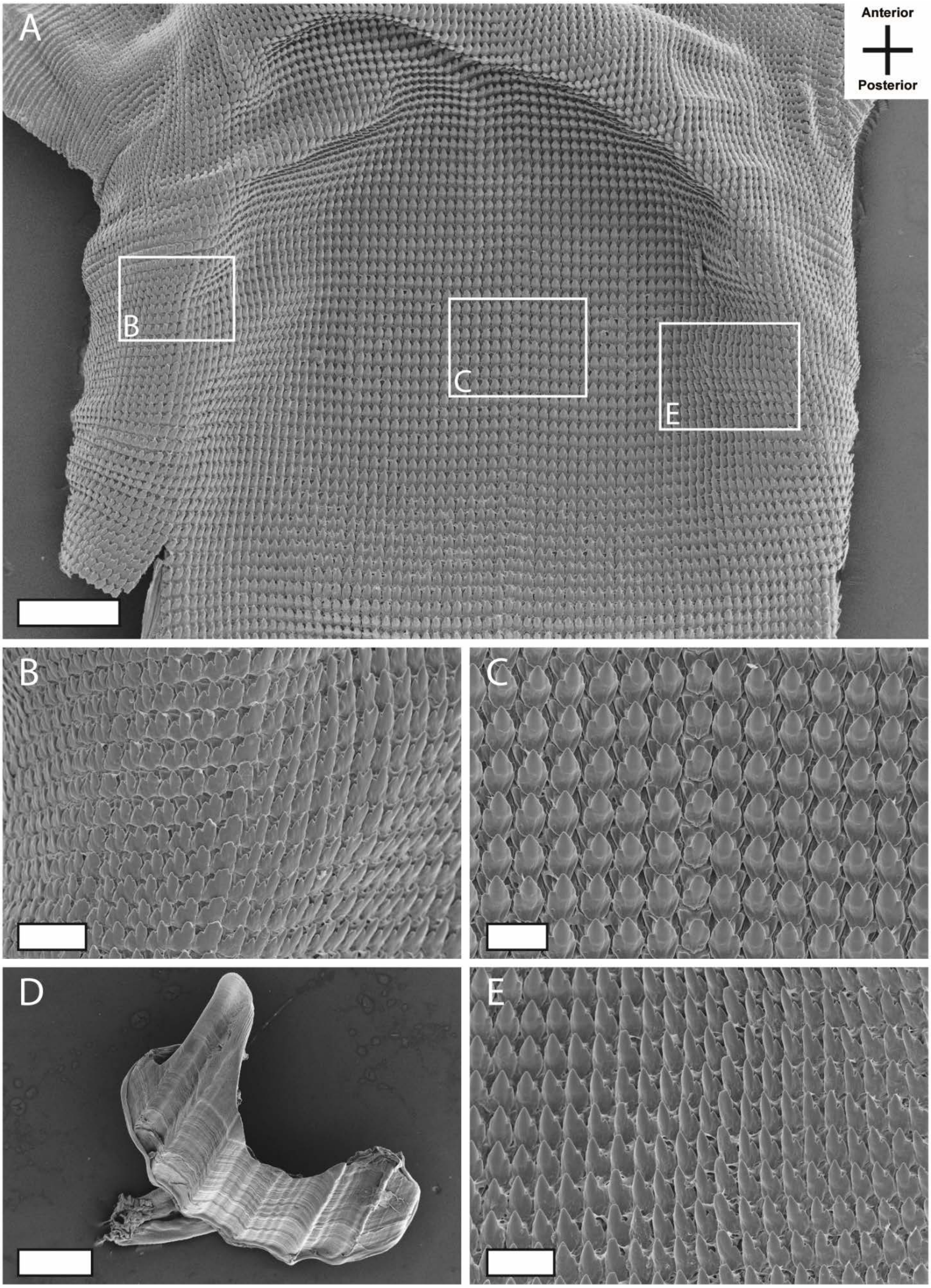

Supplementary Figure 5. SEM images of feeding organs of *Helix pomatia*. A. Radula. B. Marginal teeth. C. Central and lateral teeth. D. Jaw. E. Marginal teeth. Scale bars: A = 400 µm, B, E = 100 µm, C = 80 µm, D = 600 µm.

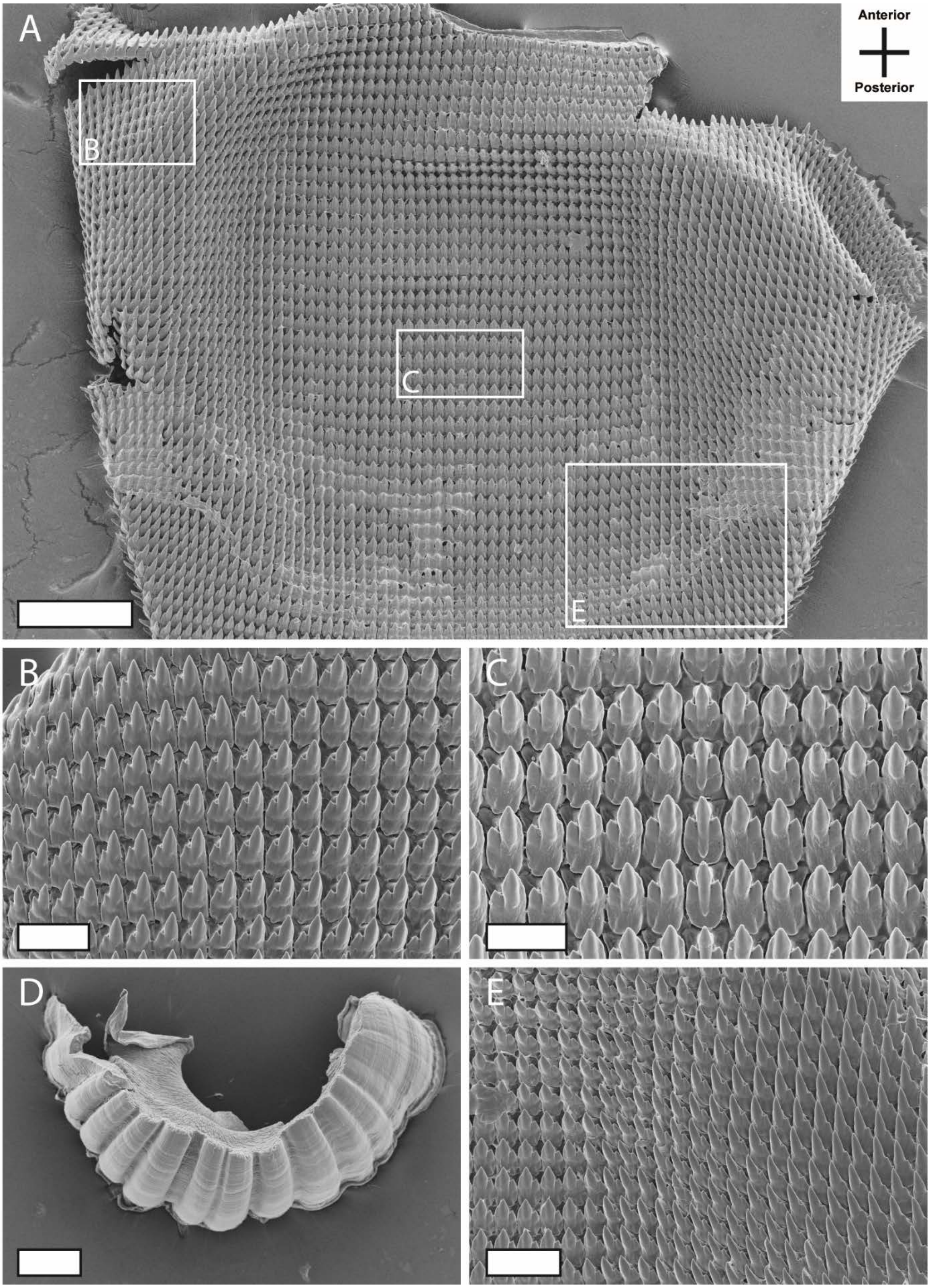

Supplementary Figure 6. SEM images of feeding organs of *Arion vulgaris*. A. Radula. B. Marginal teeth. C. Central and lateral teeth. D. Jaw. E. Marginal teeth. Scale bars: A = 300 µm, B, C = 60 µm, D = 200 µm, E = 100 µm.

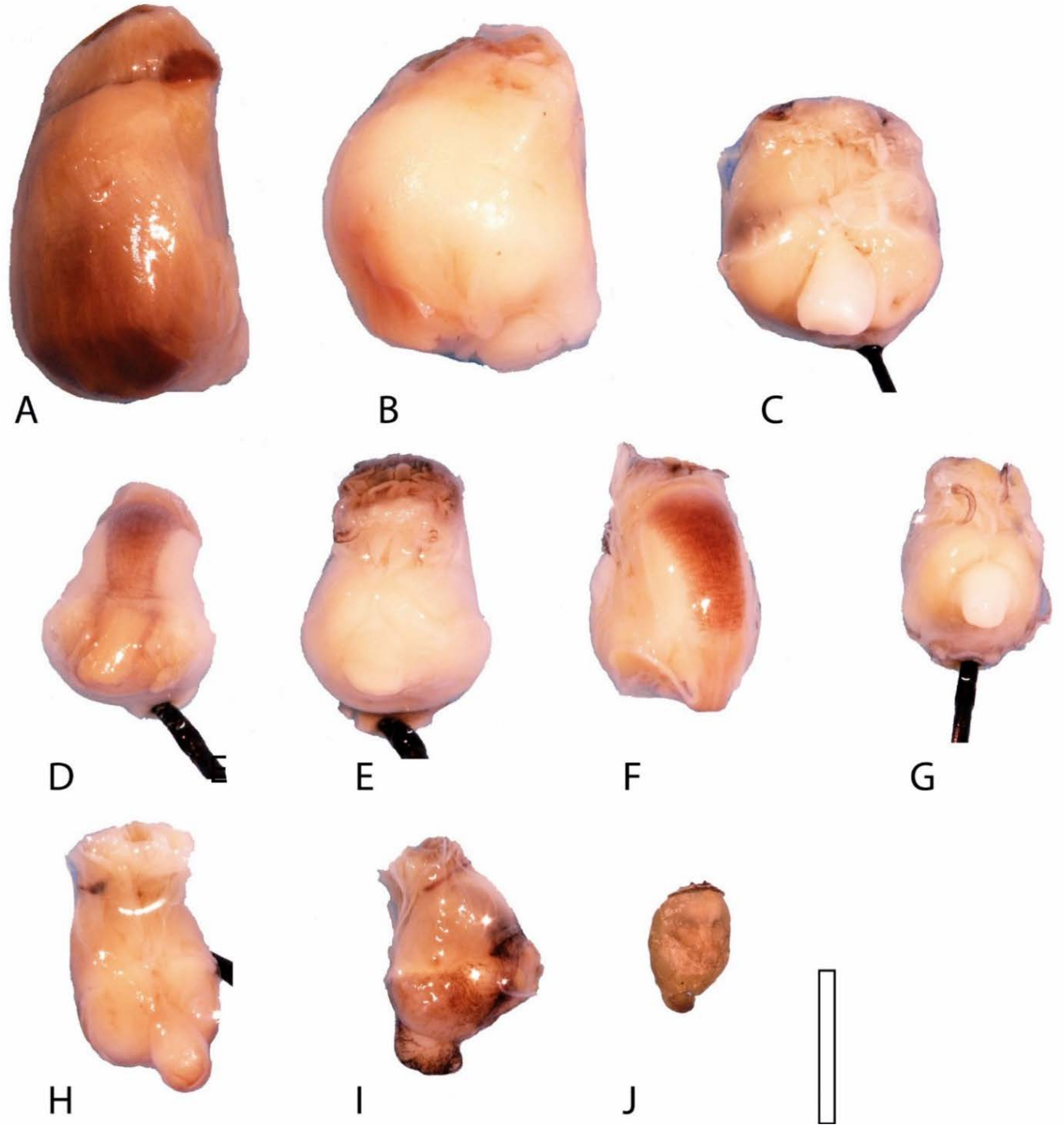

Supplementary Figure 7. Extracted buccal masses (musculature, radula, and jaw) freed from surrounding tissue. A. *Lissachatina fulica* mature no. 1. B. *Lissachatina fulica* immature no. 9. C. *Helix pomatia*. D-G. *Arion vulgaris* no. 1-4. H-I. *Cepaea nemoralis* no. 1-2. J. *Cepaea hortensis*. Scale bar = 4000 µm.

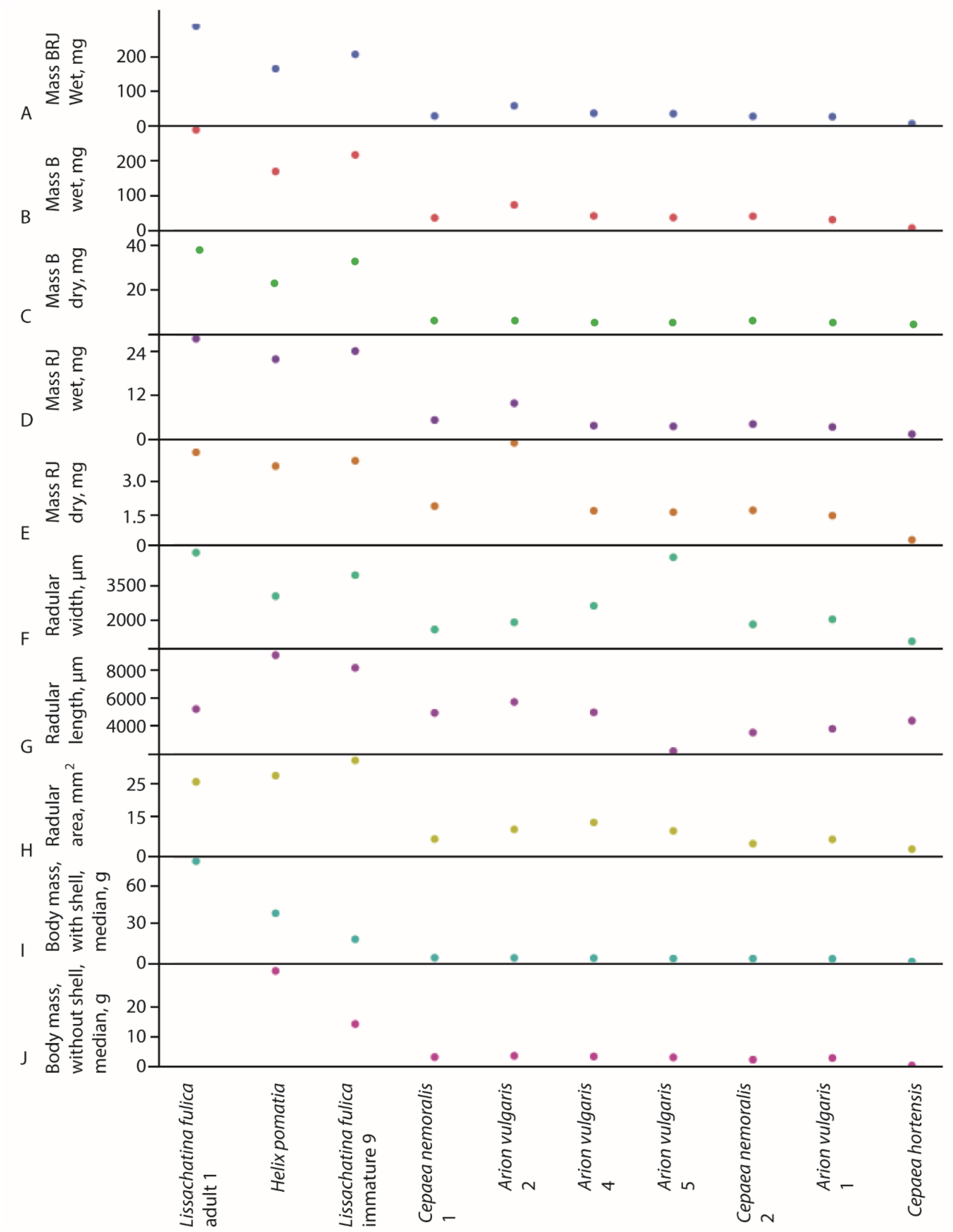

Supplementary Figure 8. From dissected specimens sorted by descending whole body mass: masses (mg) of A. wet BRJ (whole buccal mass), B. wet B, C. dry B, D. wet RJ, E. dry RJ; F. radular width (µm), G. radular length (µm), H. radular area (mm^2); I. mean body mass (g) with shell, J. mean body mass (g) without shell. For values see Supplementary Tab. 5. B, buccal mass musculature; BRJ, whole buccal mass; J, jaw; R, radula.

Supplementary Table 5. From dissected specimens (see also Supplementary Fig. 8): masses of whole buccal mass (BRJ), buccal mass musculature (B), radula and jaw (RJ); radular width, length, area; mean body mass with and without shell.

Species	Specimen	Mass BRJ	Mass B		Mass RJ		Radular			Body mass	
		wet, mg	wet, mg	dry, mg	wet, mg	dry, mg	width, µm	length, µm	area, mm²	with shell, mean, g	without shell, mean, g
Lissachatina fulica	Mature 1	286.81	290.99	38.37	27.29	4.22	4933.00	5178.00	25.54	78.00	-
Helix pomatia	Mature 1	163.30	169.43	22.86	21.69	3.62	3023.00	9077.00	27.44	38.00	31.70
Lissachatina fulica	Immature 9	205.01	217.63	33.58	23.91	3.85	3945.00	8165.00	32.21	18.00	14.10
Cepaea nemoralis	Mature 1	27.20	33.42	4.38	5.08	1.89	1558.00	4906.00	7.64	3.60	3.10
	Mature 2	26.11	38.21	4.97	3.97	1.71	1780.00	3492.00	6.22	2.95	2.25
Cepaea hortensis	Mature 1	4.92	3.73	1.64	1.21	0.43	1034.00	4351.00	4.50	0.65	0.33
Arion vulgaris	Mature 1	25.06	27.94	2.10	3.17	1.48	1999.00	3765.00	7.52	-	2.80
	Mature 2	56.66	71.52	4.45	9.64	4.63	1869.00	5701.00	10.66	-	3.50
	Mature 4	35.09	39.05	2.77	3.55	1.69	2597.00	4947.00	12.85	-	3.30
	Mature 5	33.61	34.22	2.32	3.35	1.63	4725.00	2156.00	10.19	-	3.00

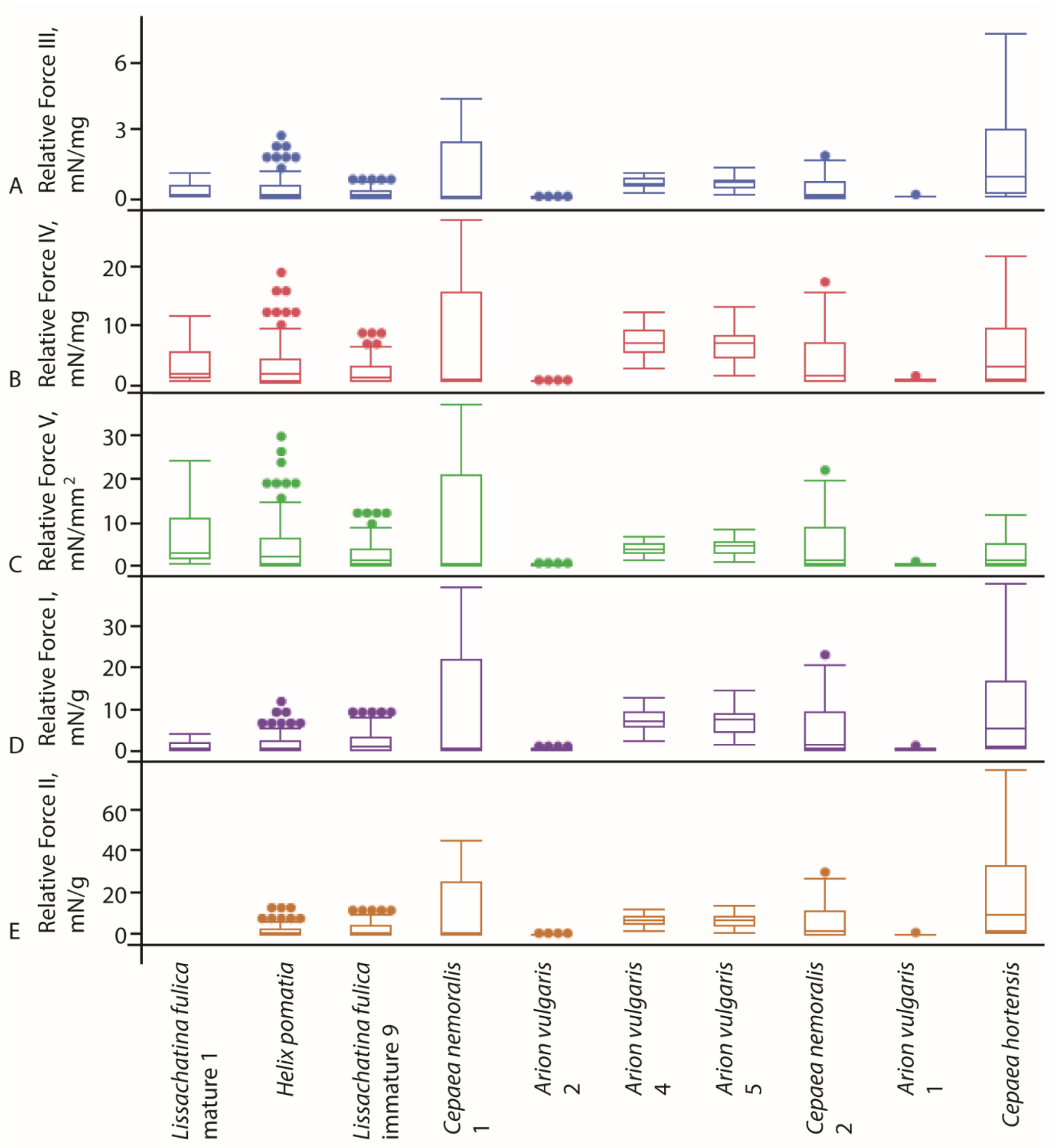

Supplementary Figure 9. From dissected specimens sorted by descending whole body mass: A. Relative Force III (force / dry buccal mass musculature, mN / mg). B. Relative Force IV (force / dry radula and jaw mass, mN / mg). C. Relative Force V (force / radular area, mN / mm^2). D. Relative Force I (force / mean body mass with shell, mN / g). E. Relative Force II (force / mean body mass without shell, mN / mg). For values see Supplementary Tab. 6.

Supplementary Table 6. From dissected specimens (see also Supplementary Fig. 9): values, mean ± SD, for Relative Force III (force / dry buccal mass musculature, mN / mg), Relative Force IV (force / dry radula and jaw mass, mN / mg), Relative Force V (force / radular area, mN / mm^2), Relative Force I (force / mean body mass with shell, mN / g), and Relative Force II (force / mean body mass without shell, mN / mg). Quantity of evaluated force measurements used for calculations. SD, standard deviation.

Species	Specimen	Relative Force III, mN/mg		Relative Force IV, mN/mg		Relative Force V, mN/mm^2		Relative Force I, mN/g		Relative Force II, mN/g		Quantity of evaluated force measurements
		**Mean**	**± SD**	**Mean**	**± SD**	**Mean**	**± SD**	**Mean**	**± SD**	**Mean**	**± SD**	
Lissachatina fulica	Mature 1	0.28	0.25	2.95	2.65	6.30e-6	5.66e-6	1.03	0.93	-	-	234
	Immature 9	0.19	0.20	1.69	1.84	2.50e-6	2.73e-6	2.24	2.44	2.86	3.12	160
Cepaea hortensis	Mature 1	1.64	1.77	5.07	5.46	2.72e-6	2.94e-6	9.43	10.17	18.57	20.03	160
Cepaea nemoralis	Mature 1	0.95	1.34	6.22	8.79	8.27e-6	1.17e-5	8.78	12.41	10.19	14.41	156
	Mature 2	0.38	0.49	3.69	4.72	4.71e-6	6.02e-6	4.96	6.35	6.50	8.32	160
Arion vulgaris	Mature 1	0.05	0.01	0.42	0.13	3.55e-7	1.09e-7	0.48	0.15	0.48	0.15	50
	Mature 2	0.02	0.01	0.16	0.04	2.85e-7	8.04e-8	0.54	0.15	0.43	0.12	60
	Mature 4	0.64	0.24	7.02	2.61	3.88e-6	1.44e-6	7.56	2.81	7.12	2.65	48
	Mature 5	0.63	0.26	6.49	2.68	4.27e-6	1.77e-6	7.24	3.00	7.24	3.00	47
Helix pomatia	Mature 1	0.33	0.39	2.58	3.01	4.08e-6	4.76e-6	1.47	1.72	1.77	2.06	270
